# The multiplex network of human diseases

**DOI:** 10.1038/s41540-019-0092-5

**Published:** 2019-04-23

**Authors:** Arda Halu, Manlio De Domenico, Alex Arenas, Amitabh Sharma

**Affiliations:** 1 Channing Division of Network Medicine, Brigham and Women’s Hospital, Harvard Medical School, Boston, MA 02115 USA; 2 Departament d’Enginyeria Informàtica i Matemàtiques, Universitat Rovira i Virgili, 43007 Tarragona, Spain

**Keywords:** Systems biology, Computational biology and bioinformatics

## Abstract

Untangling the complex interplay between phenotype and genotype is crucial to the effective characterization and subtyping of diseases. Here we build and analyze the multiplex network of 779 human diseases, which consists of a genotype-based layer and a phenotype-based layer. We show that diseases with common genetic constituents tend to share symptoms, and uncover how phenotype information helps boost genotype information. Moreover, we offer a flexible classification of diseases that considers their molecular underpinnings alongside their clinical manifestations. We detect cohesive groups of diseases that have high intra-group similarity at both the molecular and the phenotypic level. Inspecting these disease communities, we demonstrate the underlying pathways that connect diseases mechanistically. We observe monogenic disorders grouped together with complex diseases for which they increase the risk factor. We propose potentially new disease associations that arise as a unique feature of the information flow within and across the two layers.

## Precision medicine: multi-layered map of diseases enables new disease classification

The joint analysis of shared genes and symptoms on a multi-layered disease network uncovers an alternative grouping of diseases. An international team of researchers from Brigham and Women’s Hospital and Universitat Rovira i Virgili built and analyzed a large-scale network of diseases that consisted of two layers representing gene and symptom relationships. On a global scale, diseases that shared genes also tended to share symptoms. An algorithm specifically designed to identify patterns in multi-layered networks determined groups of diseases that were highly similar to each other both genetically and symptomatically. This approach holds the potential to transcend today’s clinical observation-based disease classification systems. It may pave the way for a molecular-based disease classification, the discovery of novel disease relationships, and ultimately personalized diagnosis and treatment.

## Introduction

The advent of next-generation sequencing (NGS) and genome-wide association studies (GWAS) has led to the accumulation of a vast amount of disease-gene associations.^1^ In addition, high-throughput experimental studies and proteomic technologies have resulted in extensive protein interactions maps. Connecting disease-related phenotypes to their underlying molecular mechanisms and genetic constituents is crucial for a better understanding of complex human diseases. The emerging field of network medicine offers the tools of network science for distilling relevant insight from the growing sets of molecular disease omics data.^2^ One of the earliest attempts at exploring the higher-level implications of disease-gene associations from the network perspective was the construction of genotype-based disease networks, useful to show the global organization of diseases around functional modules^3^ and to infer comorbidity relations between diseases.^4^ On the same basis, phenotypic–based disease networks were constructed by text-mining large-scale Medicare data, systematically classifying diseases based on phenotype similarity,^5^ and facilitating the identification of patterns of disease progression.^6^ Since these pioneering works, many studies have focused on adding to the growing compendium of disease-disease associations. For example, Suratanee et al. identified disease-disease associations using a scoring method based on random walk prioritization in the protein-protein interaction network and identified novel disease-disease interactions.^7^ Yang et al. measured disease similarity based on differential coexpression analysis to elucidate dysfunctional regulatory mechanisms and arrived at novel interactions between diseases, whose shared molecular mechanisms have recently been uncovered.^8^ Menche et al. identified common mechanistic pathways between diseases by the overlap of disease modules.^9^

Despite these large-scale efforts, the characterization of human disease is incomplete if a single source of information, whether molecular or clinical, is considered in isolation owing to the deeply entangled and causal nature of these different types of data. To address this aspect, researchers have started exploring disease associations using multiple data sources. In a recent study, Zitnik et al. applied a matrix factorization based data fusion approach on different molecular and ontological data that resulted in a multi-level hierarchy of disease classification and predicted previously unknown disease-disease associations.^10^ In a similar vein, Moni et al. developed a multiplex network model, which combines patient-specific diagnostics, integrative omics, and clinical data to generate comorbidity profiles of diseases to help stratify patients and potentially derive personalized medicine solutions in the future.^11^ More recently, Klimek et al. used different molecular mechanisms, genetic and environmental, to define the layers of multiplex comorbidity networks.^12^ Cheng et al. presented a method that simultaneously uses functional and semantic associations to calculate the similarity between pairs of diseases and predict new associations.^13^ Sun et al. developed a combined similarity score using annotation-based, function-based, and topology-based disease similarity measures; compared their predictions against genome-wide association studies; and predicted novel disease associations.^14^

The idea of incorporating multiple sources of information finds its direct counterpart in the literature of multiplex and multilayered networks^15^ where the structure and dynamics of various social, technological and biological networks^16–23^ have been shown to be better understood in terms of multiple interconnected layers of networks.^24–31^ Inspired by this prior work, we hypothesize that we should be able to uncover novel information about disease-disease relationships by incorporating genotypic and phenotypic information simultaneously. To this end, we build a multiplex disease network consisting of a genotype-based layer and a phenotype-based layer. We then find multiplex communities of diseases on this multiplex network using a community detection method recently developed by us, multiplex Infomap,^32^ to uncover associations between seemingly disparate diseases that have common molecular mechanisms.

The development of a molecular-based disease classification that links genotype and phenotype layers is remarkably challenging and currently remains an unresolved problem. Our approach helps resolve this issue by offering an in-depth understanding of cohesive multiplex disease communities. In the age of the proliferation of high-throughput omics methods, disease classification based only on clinical traits and pathological examination is insufficient by itself and may be misleading.^33^ The inclusion of multi-omics information in molecular-level disease-disease relations is expected to improve disease classification.^33^ Some of the recent disease classification efforts focused on probabilistic clustering algorithms. Hamaneh et al. devised a cosine-similarity approach based on information flow on disease-protein networks, which outputs clusters of similar diseases.^34,35^ The grouping of diseases based on their temporal progression (disease trajectory) is another important aspect of disease classification as it presents us with the possibility of predicting future diseases given the patient’s history. Jensen et al. studied the progression of diseases using large-scale health registry data and identified significant classes of trajectories as well as teased out the key diagnoses central to these trajectories.^36^ Furthermore, it has been observed that Mendelian disorders often predispose patients to complex disorders. In this respect, an important linkage has been identified regarding the reconciliation of Mendelian and complex disorders, where Blair et al. mined medical record data to infer associations between these two types of disorders and uniquely mapped each complex disorder to a collection of Mendelian disorders.^37^ In light of such recent developments, we focus on disease classification as a primary application of our approach. We, therefore, deploy our information flow compression community detection technique on the multiplex network of diseases for a proof-of-concept study on disease classification, and analyze our disease communities for biological relevance and novelty. Overall, our study represents a novel addition to the body of works addressing this subject in two ways: (1) We provide a large-scale multiplex disease network, which has not previously been constructed consistently using multiple aligned data sources; and (2) We apply, for the first time, a multiplex community detection method on a global disease network for the purposes of disease classification, in contrast to other similar methods that have been used on multilayered molecular networks to determine functional similarities between biological molecules.

## Results

### Disease-disease interactions shared by genotype and phenotype layers

**Fig. 1:**
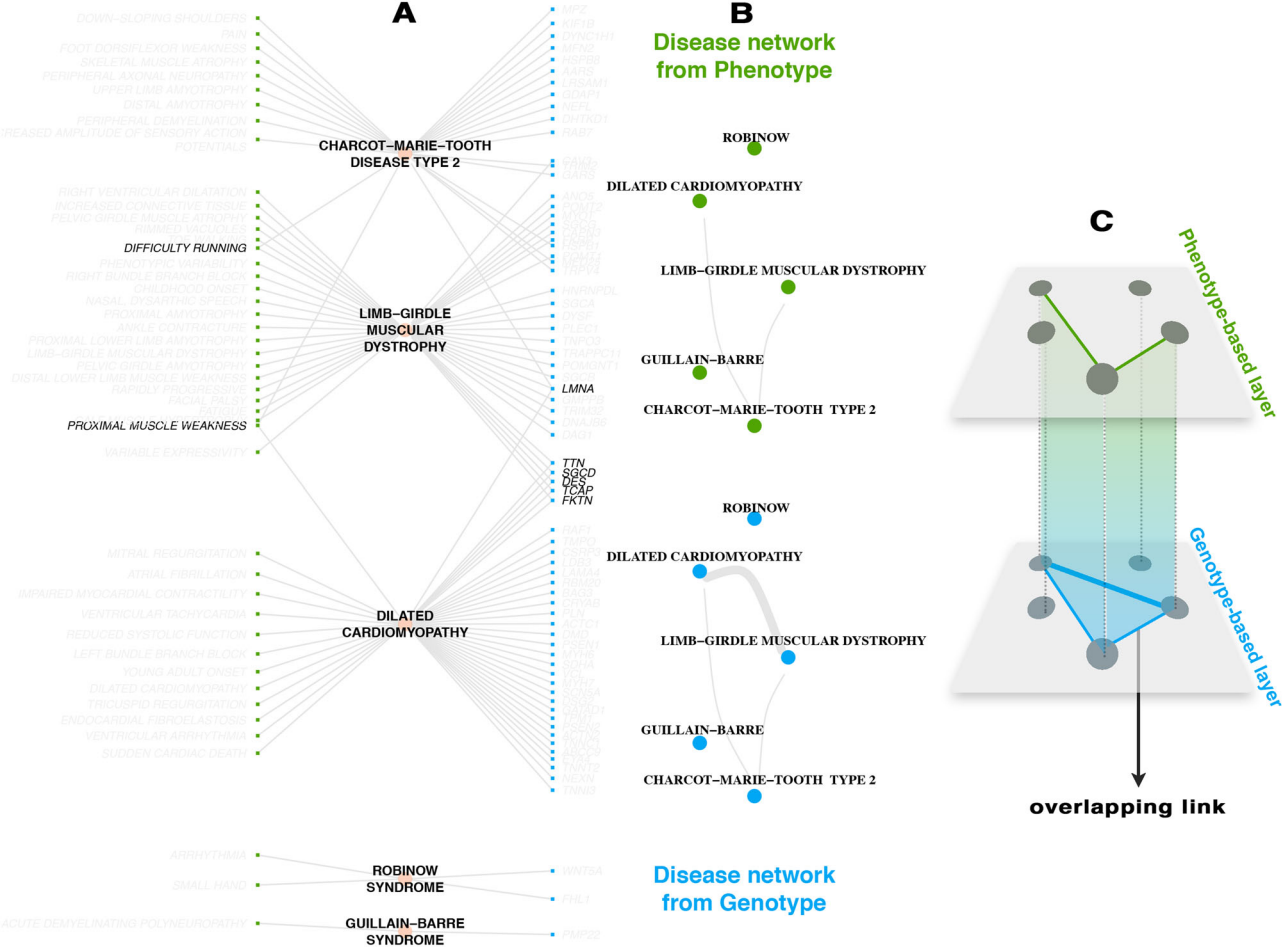
The multiplex disease network. **a** Tripartite network of symptoms (green nodes on the left), diseases (pink nodes in the middle) and genes (blue nodes on the right). Symptoms and genes that are shared between diseases are shown in darker text. **b** Phenotype- and genotype-based disease-disease networks where diseases are connected in the genotype layer (blue) if they share at least one gene and connected in the phenotype layer (green) if they share at least one symptom. The thickness of the edge is proportional to the number of common genes or symptoms. **c** The two networks are considered as layers of a multiplex system, where nodes are the diseases and colored links encode their interactions. Disease-disease interactions that are present in both layers are denoted “overlapping links.”

We used two different data sources concerning genotypic and phenotypic information about diseases (see Methods for details). Gene-disease relationships are used to build the genotype-based disease-disease interaction network layer, where two diseases are linked if they share a common disease gene. Symptom-disease relationships are used to build the phenotype-based disease-disease network, where two diseases are linked if they share a common symptom. In Fig. 1 we show a sketch of this procedure applied on a sub-sample of the combined dataset. The multilayer structure is obtained by considering the two networks as layers of a multiplex system in which a disease has at least one connection in at least one layer. The same set of diseases are represented in both layers and diseases with no connections in one layer are represented as isolated nodes in that layer. The final multiplex network thus consists of 779 non-isolated diseases with 1115 genotypic and 5005 phenotypic relationships (see Methods for further details and for the analysis of gene similarity of disease pairs).

To obtain initial insights about the common genetic and phenotypic mechanisms underlying our disease networks, we considered the shared edges between the genotype and phenotype layer simply when the two network layers are overlaid, i.e., disease pairs that have at least one gene and one symptom in common. We found a highly significant enrichment of coinciding disease-disease interactions across layers with 139 overlapping edges (see Methods, SI Section 1 and Figs. S1 and S2), which reveals that diseases that have common genes also tend to share symptoms. Indeed, a closer look at these overlapping edges reveals that they connect disorders that are variable expressions of the same mutation such as MASA syndrome and X-linked hydrocephalus, diseases related to the same gene with important overlapping clinical features, such as cerebreal amyloid angiopathy and Alzheimer disease; similar but milder disorders caused by the same gene, such as Roberts Syndrome and SC Phocomelia Syndrome; subtypes of a disease, such as Gilbert’s Syndrome and Crigler-Najjar Syndrome; as well as subtler associations, such as scapuloperoneal myopathy and hypertrophic cardiomyopathy, bronchiectasis and cystic fibrosis, type 2 diabetes mellitus and maturity-onset diabetes of the young, and Noonan syndrome and juvenile myelomonocytic leukemia. It is also interesting to note that on the network-topological level, the different levels of granularity and the distinct local clustering of the two networks (Figs. S3 and S4) result in the heterogeneous distribution of overlap links around what may be called “overlap hubs” (Fig. S5). We find that these hubs are either diseases defined as groups of diseases or multi-system diseases that affect a number of organs and have wide ranging symptoms as well as common genetic factors with other diseases (see SI Section 2).

### Single-layer disease communities

After building the disease multiplex and probing its structural characteristics, we focus on its higher-level organization. While the simultaneous representation of diseases in the genotype and phenotype space is a very informative abstraction by itself, it is difficult to investigate the interactions between all 779 diseases at once. We, therefore, attempt to decode it further into biologically cohesive disease communities. First, we studied the community partition in each layer separately, i.e., without exploiting the available multiplex information. We identified the communities by using Infomap, a well-known algorithm based on the compression of information flow.^38,39^ We selected this method among a number of other community detection methods since it has a direct generalization for multiplex networks (see Fig. S6 and SI Section 3 for a discussion on the choice of community detection technique). As the community detection algorithm does not label communities in any particular way, i.e., it is blind to the underlying biology, we check for the disease overlap between all possible pairs of communities in the genotype and phenotype layers, separately. The overlap is quantified by calculating the average Jaccard index for the disease overlap between all pairs of communities. To check the statistical significance of the index obtained, we compare it to a randomized background where the topological aspects of the two networks are conserved. To ensure that topologies are comparable, we randomize the network in each layer by keeping the degree distribution constant, using degree-preserving randomization (SI Section 4). Remarkably, we find that the communities into which the algorithm puts the diseases are layer specific, meaning that there is little correspondence between the disease communities in the two layers. The average Jaccard index for the disease overlap between layers is 〈*J*〉 = 0.00286, which is indistinguishable (*z*-score = 0.882) from that of the degree-preserving randomized layers where the mean of the average Jaccard distribution of the randomized ensemble is 〈*J*〉 = 0.00261 ± 0.000289 (Fig. S7). This indicates that the Infomap algorithm, when applied to genotype and phenotype layers separately, results in distinct disease groupings specific to the underlying network, owing to the different kinds of information encoded in each layer. However, we argue that disease communities in each layer are meaningful from two different aspects, no matter how distinct they are. In the genotype layer, diseases in the same community represent diseases with common molecular roots, whereas the diseases in the same community in the phenotype layer have similar clinical manifestations. Rather than conflicting pieces of information, we regard these as two complementary sources of information that have to be reconciled using multiplex networks.

### Multiplex disease communities

We further analyzed the human disease multiplex network to shed light on the functional organization of diseases when genotypic and phenotypic information are considered simultaneously (see Methods). Using the Multiplex Infomap algorithm, we identified multiplex disease communities with the aim of grouping together diseases based on their similarity at both the molecular and symptomatic levels. To assess the cohesiveness of the multiplex disease communities, we look for similarities between disease pairs within communities. Our hypothesis is that if two diseases in a community share clinical characteristics, then they should have common biological pathways and genes. To test this hypothesis, we calculated biological process similarity, comorbidity, gene overlap, and phenotype semantic similarity for each disease pair in each multiplex community. Given that our multiplex consists of a genotype and phenotype layer, we selected these four criteria such that both molecular/genotypic and clinical/phenotypic factors are accounted for. Our goal is to show that multiplex communities are able to capture known disease-disease relationships successfully, as well as offer new insights into unknown disease relationships. The direct quantitative comparison of the contents of communities is a well-known challenge,^40^ which is compounded in the case of single and multilayer networks. Our approach, by comparison, provides us with a platform where the general biological cohesiveness of communities determined within single layers can be compared quantitatively and consistently to the communities determined in the multiplex network through these four measures.

Our corpus of 779 OMIM diseases, consisting of complex as well as monogenic disorders, is divided into 128 multiplex communities, with sizes ranging between 2 and 91 diseases (see Supplementary Table 1 for the size of each community). The number of unique diseases classified into groups saturates quickly as we proceed cumulatively from the largest group to the smallest group (see SI Section 5, Fig. S8). Here, for statistical evaluation, we concentrate on those communities consisting of at least 10 diseases.

**Fig. 2:**
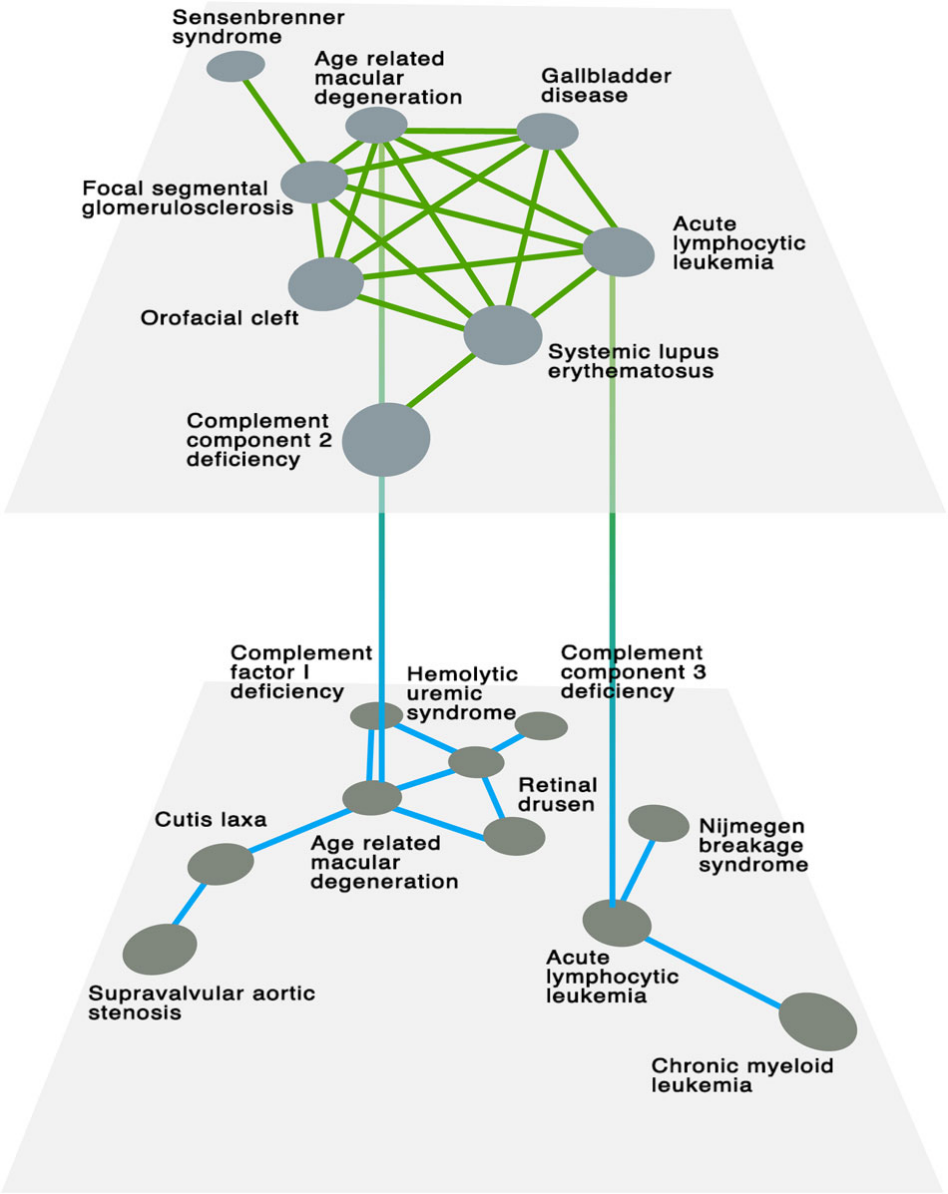
Multiplex communities. An emblematic example of multiplex community bridging genotypic and phenotypic information to discover new disease-disease interactions that, otherwise, would not be identified from standard analysis. In this case (Multiplex Community 15) there are no edges in common across the two layers (i.e., there is no phenotype interaction with a genetic explanation) and only two diseases are shared by the communities in the two layers, i.e. Age-related macular degeneration and acute lymphocytic leukemia, due to acute leukemia being associated with ocular comorbidity

It is worth noting two unique properties of our multiplex community detection method: namely, a disease can be assigned (i) to the same community twice, i.e., it belongs to the same community across the two layers; and (ii) to two different communities. In the first case, the community structure can be distinguished between the two layers such that one can state that a disease belongs to the same disease community in both the genotype and the phenotype layers. The second case is especially important for our purposes because it defies the current clinical observation-based disease classification and allows for a more organic classification with more flexible boundaries that respect the molecular underpinnings of diseases. In our dataset, 215 diseases act as bridges between genetic and phenotypic communities resulting from the overlapping of multiplex communities (see SI Section 6, Figs. S9 and S10). Overall, these communities represent multiplex structures across layers that bring together pathobiological elements that are not identifiable from the analysis of each layer separately (Fig. 2). For example, the multiplex community shown in Fig. 2 has no common links between the two layers; however, the two layers are brought together by two diseases, age-related macular degeneration and acute lymphocytic leukemia, owing to the fact that acute leukemia is associated with ocular comorbidity.^41^ Furthermore, this connection brings together the two disconnected parts of the genotype layer and offers an insight into the molecular ties between these disconnected diseases, such as that in which complement component 3 (C3) has been suggested as a biomarker for Nijmegen Breakage Syndrome (NBS).^42^

**Fig. 3:**
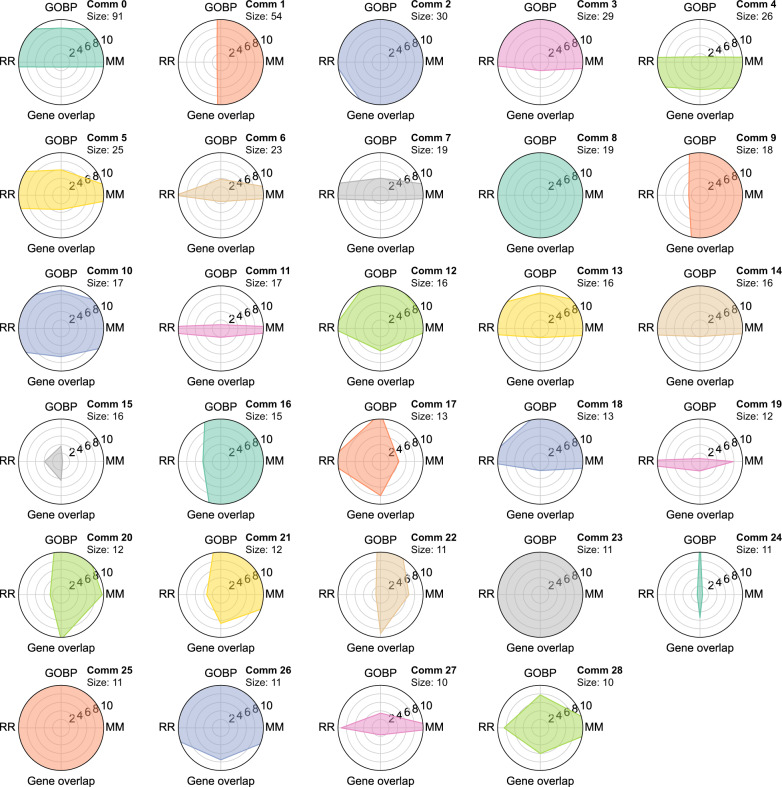
Similarity assessment of multiplex disease communities. Radar plots of the 29 disease communities with size 10 or more, showing the −log *P*-values as the concentric circles. The molecular similarity represented by the North-South axis is for Gene Ontology:Biological Process (GO:BP) and Gene Overlap, whereas phenotypic similarity represented by the East-West axis is for relative risk (RR) comorbidity and MimMiner (MM) phenotype semantic similarity. The larger the overall shaded area, the more significant the intra-community similarity. Points confined to the innermost circle (−log *P*-value < 2, or *P*-value > 0.01) represent non-significant intra-community similarities for the respective similarity measure

As a general survey of all the communities, we first map the pairwise similarities of the diseases in all communities in a heatmap where we order the communities in order of decreasing size (see SI Section 7, Figs. S11–S14). For each community, we assess the significance of the intra-community similarity according to the four metrics previously introduced by comparing the distributions of the community values against the background of all disease pairs in all communities (see Fig. S15 for details). For the 29 largest communities with size greater than or equal to 10, we find that the majority of the communities have significantly high similarity. In particular, the number of communities that have significantly high similarity is 26/29 for comorbidity measured in terms of relative risk (RR), 23/29 for gene overlap measured by Jaccard index, 20/29 for GO: Biological Process similarity, and 27/29 for semantic similarity measured by MimMiner. Furthermore, 14/29 clusters have significantly high similarity with respect to all of the four similarity measures, and 29/29 clusters have significantly high similarity by at least two of the four similarity measures (Fig. 3). Taken together, this analysis shows that owing to the fusion of information on both layers of the disease multiplex, our multiplex disease communities are biologically cohesive at both genetic and phenotypic levels.

Using an external validation dataset of disease-disease interactions,^43^ we measured our disease communities’ potential to host novel disease-disease interactions (SI Section 8). In terms of the number of potentially novel edges evidenced by Disease-Connect, multiplex disease communities outperformed genotype and phenotype communities (Fig. S16). This result demonstrates the ability of multiplex communities to uncover novel or underappreciated molecular disease associations, owing to the ability of multiplex communities to bridge different communities that are separately represented in single-layers.

As a further validation of the multiplex disease communities, we verify that they are distinct from randomized communities in that they have significantly higher similarity than random according to the four similarity measures (SI Section 9). More importantly, to test the non-additive cooperative effect of multiplexity and to give a meaningful background against which the cohesiveness of multiplex disease communities can be compared, we carried out the same molecular and phenotypic similarity assessment on genotype- and phenotype-layer-specific disease communities (SI Section 10). We found that, simply in terms of performing dichotomously as “better or worse than,” (i) multiplex communities perform better than the genotype-layer communities in terms of phenotypic similarity measures, and (ii) multiplex communities perform better than the phenotype-layer communities in terms of the molecular similarity measures while performing comparably with or better than the phenotype layer communities in terms of the phenotype similarity measures (Fig. S17). We note that, given the lack of overlap of single-layer communities, it is not surprising for phenotype-based layers to do worse in terms of genetic similarity measures, and vice versa. Nevertheless, with multiplex communities scoring slightly better than single-layer communities overall, this finding may suggest that the multiplex disease network is, indeed, “greater than the sum of its parts,” compensating for the lacking features of each single layer and reflecting an all-around cohesive picture rather than a more limited view of one of the two aspects.

### Confirming established disease associations and finding new ones

We next proceeded to the disease-level and investigate disease relationships in multiplex communities for new biological insight. Our aim is two-fold: first, verify known disease relationships, showing that the communities are reliable; and second, uncover novel disease-disease relationships. To this end, we first select disease pairs that are expected from known literature associations. We calculated the co-occurrence of diseases in the entire PubMed database using the PubAtlas query tool (see Methods) and built a “publication co-occurrence network” of diseases in the multiplex community. This construct helps us to identify the disease pairs with high co-occurrence, which we would expect to see in the same disease community based on prior research, as opposed to possibly novel disease pairs with few literature co-occurences.

For example, well-established connections are found in Multiplex Community 23 consisting mainly of rare, inherited skeletal abnormalities (Fig. S18). Such connections are (Fig. S18e) the Hunter-Thompson and Grebe types of acromesomelic dysplasia, both of which are caused by a mutation in the GDF5 gene. Similarly, tarsal-carpal coalition syndrome and proximal symphalangism, which are both caused by a heterozygous mutation in the NOG gene, have high co-occurrence in the literature. The close relationship of these expected pairs of diseases are further evidenced by their high comorbidity, high biological process similarity, and high phenotype semantic similarity. Multiple synostoses syndrome also has high publication co-occurrence with tarsal-carpal coalition syndrome and proximal symphalangism, with which it shares one of its three related genes. It also has high biological process similarity and moderate phenotype semantic similarity with those two diseases. In addition to these already established relationships, our multiplex disease communities also bring together many diseases with subtler connections. For example, fibular hypoplasia and complex brachydactyly are also closely associated with acromesomelic dysplasia types, and arise from a mutation in the same gene, GDF5, even though these diseases do not appear together in the literature and have low comorbidity. Furthermore, they have high biological process similarity and moderate semantic similarity with acromesomelic dysplasia types. Likewise, even though synpolydactyly and acrocapitofemoral dysplasia do not have any shared genes, they are grouped together owing to their high comorbidity and similar Gene Ontology (GO) biological process terms. Given that acrocapitofemoral dysplasia is a recently delineated skeletal dysplasia characterized by brachydactyly, synpolydactyly, which is another digit dysplasia, might have common underlying molecular mechanisms with this rare disease. These new connections show that our multiplex disease communities are able to capture the molecular basis of connections between diseases even if they have not been associated with each other simply by their clinical manifestations.

**Fig. 4:**
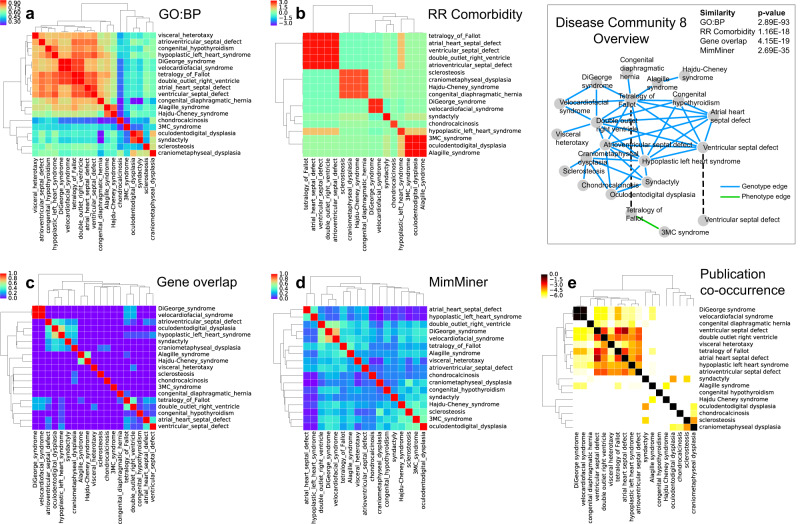
Anatomy of Multiplex disease community 8. Disease community 8 is characterized by rare congenital heart defects and skeletal anomalies. **a** GO: Biological Process similarity heatmap, where similarity scores range between 0 and 1. **b** Relative risk (RR) comorbidity heatmap, where the colors represent the logarithm of the RR values. **c** Gene overlap, quantified by the Jaccard index. **d** MimMiner phenotype semantic similarity heatmap, where similarity scores range between 0 and 1. **e** PubMed literature co-occurrence heatmap and representative network, where the number in each cell denotes the number of publications that have the co-occurence of the queried keywords. The color of each cell represents the Jaccard index; a redder cell means higher literature co-occurrence weighted by the total number of publications

**Fig. 5:**
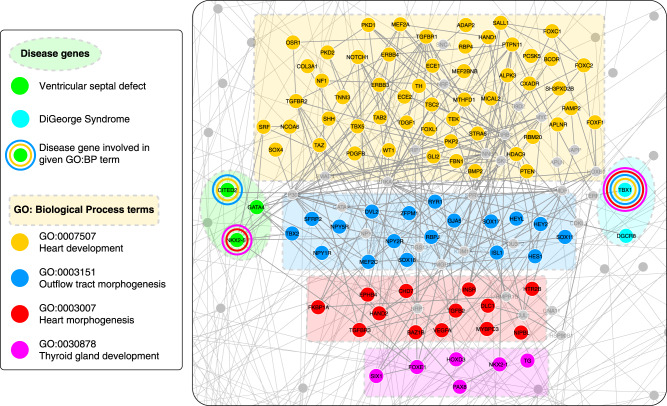
Biological process similarity of diseases. Portion of the human protein-protein interaction (PPI) network depicting the localization of disease genes and genes related to relevant biological processes. Nodes represent protein-encoding genes and edges represent literature-documented physical interactions between them. Disease genes (denoted with the dashed ellipse areas) associated with ventricular septal defect (green nodes) and DiGeorge Syndrome (cyan nodes) are connected by biological processes related to cardiac development (nodes related to outflow tract morphogenesis in blue; heart morphogenesis in red; heart development in yellow) and endocrine development (nodes related to thyroid gland development in magenta). Concentric circles around disease genes indicate overlap of disease genes with the biological process of that color

Among the many interesting multiplex disease communities (see Supplementary Table 1 for a full list), Multiplex Community 8 groups together rare skeletal abnormalities with rare congenital heart defects, which usually have related clinical manifestations as well as shared genetic causes (Fig. 4). DiGeorge syndrome and velocardiofacial syndrome have traditionally been studied together in the literature, and both are caused by a hemizygous deletion of chromosome 22q11.2 and are also believed to be caused by point mutations on the TBX1 gene. Tetralogy of Fallot, by contrast, is clinically associated with four types of atrial and ventricular defects: atrial septal defect, ventricular septal defect, double outlet right ventricle, and atrioventricular canal defect, classified with the same ICD9 code (745). These diseases also share many genetic elements resulting in their being clustered together in terms of gene overlap (Fig. 4c). Overall, we see that DiGeorge syndrome/velocardiofacial syndrome and the cardiac defects related to Tetralogy of Fallot all have very high GO Biological Process similarity despite not sharing many genetic factors (Fig. 4a). When we look closer at two of the diseases in this group, namely DiGeorge syndrome and ventricular septal defect, at the molecular level, we see that biological processes related to cardiac development such as outflow tract morphogenesis (GO: 0003151), heart morphogenesis (GO: 0003007), heart development(GO: 0007507), as well as processes related to endocrine development, such as thyroid gland development (GO: 0030878), are shared between the genes of these two diseases, underpinning the biological similarity of the shared symptoms of these diseases. From a developmental biology perspective, these disorders, in part, reflect dysmorphogenesis at the branchial cleft level, which suggests that developmental defects may be driven by otherwise “Mendelian” mutations in a unique (temporal) developmental biology context. To assess the significance of the biological similarity between these disease genes and the related biological process, we calculate the biological process similarity (see Methods) of randomly selected genes from the interactome with each of the above processes. As expected, both diseases have significantly higher biological process similarity with outflow tract morphogenesis (*S*_*GOBP*_ = 0.687, *z*-score = 2.57 and *S*_*GOBP*_ = 0.747, *z*-score = 2.87 for ventricular septal defect and DiGeorge syndrome, respectively), heart morphogenesis (*S*_*GOBP*_ = 0.686, *z*-score = 2.45 and *S*_*GOBP*_ = 0.746, *z*-score = 2.72 for ventricular septal defect and DiGeorge syndrome, respectively), heart development (*S*_*GOBP*_ = 0.676, *z*-score = 2.17 and *S*_*GOBP*_ = 0.727, *z*-score = 2.41 for ventricular septal defect and DiGeorge syndrome, respectively) and thyroid gland development (*S*_*GOBP*_ = 0.656, *z*-score = 2.23 and *S*_*GOBP*_ = 0.717, *z*-score = 2.65 for ventricular septal defect and DiGeorge syndrome, respectively), compared to random expectation (Fig. 5). The other important disease group in this community consists of sclerosteosis, craniometaphyseal dysplasia, oculodentodigital dysplasia, syndactyly, chondrocalcinosis 3MC syndrome, and Hajdu-Cheney syndrome, which are all anomalies of the bones. Hajdu-Cheney syndrome, in particular, has many cardiovascular manifestations, including atrial and ventricular septal defects, and is, hence, effectively the common disease that bridges the cardiovascular and bone related diseases in this disease community.

As an additional check to verify the reliability of the boundaries of the multiplex disease communities, we compare the intra-community publication co-occurrences with inter-community publication occurrences to test whether the high publication co-occurrence of intra-layer disease pairs is accompanied by a lack of publication co-occurrence for inter-layer disease pairs. We found that the intra-community publication co-occurrence was higher than the inter-community case for nearly all communities (SI Section 11, Figs. S19–S23).

Smaller communities with five or fewer diseases contain a smaller fraction of the overall diseases, with 72 communities comprising 146 diseases. A quick look at some of the many smaller communities reveals that these communities are mostly completely homogeneous, consisting of synonymous diseases, Mendelian diseases and their direct complications, subtypes of the same disease, or diseases with close genetic roots (see Supplementary Table 1). They nevertheless offer very interesting disease associations that are novel. For example, we find that idiopathic pulmonary fibrosis (IPF) is grouped together with common variable immunodeficiency and immunoglobulin alpha (IgA) deficiency. Indeed, IgA in serum has recently been proposed as a prognostic biomarker for IPF.^44^ While space limitations preclude our discussing every disease community in detail, given the high cohesiveness of multiplex disease communities, readers can draw similar possibly interesting disease-disease interactions from any of the communities using Supplementary Table 1 as reference.

Finally, for a complete picture of disease-genotype relations, we have carried out the same analysis on the disease multiplex constructed using GWAS data for the genotype-layer instead of using OMIM (see SI Section 12). Our results show that, once again, despite the distinct sizes and topologies of the GWAS and OMIM disease-gene networks,^45^ we are able to capture cohesive disease groups. As an example, disease community 1 (Fig. S24) brings together various types of amyotrophic lateral sclerosis (ALS) with a group of cancer subtypes. The association between ALS and cancer has recently been suggested, where significantly elevated risk of ALS death among survivors of melanoma has been shown.^46^

These proof-of-concept examples show that the multiplex network of human diseases, coupled with community detection methods designed to highlight the interplay between different layers of the network, captures the biological similarity implicitly in different data sources. It, therefore, brings a new dimension to disease relationships that cannot be achieved if we study disease networks from the genotype and phenotype aspects separately.

## Discussion

To obtain an in-depth understanding of the molecular basis of human disease, we need to recognize the complex relationship between genotype and phenotype in response to environmental and genetic influences. At the current time, one of the most important factors hampering the effective characterization of diseases is the lack of apparent connection between how they manifest and what their molecular underpinnings are. With this issue as the starting point of this study, we made an attempt to analyze systematically the structural and functional aspects of the multiplex network of human diseases. We showed that diseases with common genes tend to share symptoms, and we uncovered the complementary nature of genotype and phenotype by examining the interplay between the two network layers. We reported our finding that, through the representation of disease relationships in a multiplex network, a wide range of monogenic and complex diseases can be grouped into communities that are cohesive at both molecular and symptomatic levels. We argued that the disease communities found in this way offer a flexible description of pathobiological processes that melds together genotypic and phenotypic aspects, while community detection applied on separate layers results in layer-specific and distinct, yet complementary, disease groupings. Disease networks have often been studied in the context of either genetic relationships between diseases, i.e., shared genes,^3^ or phenotypic relationships between diseases, i.e., comorbidity^6^ and shared symptoms.^47^ Yet, it is crucial to recognize that genotype and phenotype maps offer distinct and complementary views of biological systems.^48^ We, therefore, believe that showing this complementary nature of genotype and phenotype by simultaneously considering both types of connections on a multiplex disease network that has specifically been constructed using data collected and filtered in a consistent manner for both layers provides important insights that were previously elusive. In our view, this picture of complementary results obtained on single layers is important to recognize as a current limitation that can be overcome with multiplex network methods. In addition, we find it important to note the inherent effect of the current incompleteness of the genotype- and phenotype-layers, which stems from many yet-unknown disease-gene or disease-symptom relationships. While we demonstrated the advantage of the multiplex disease network in its present form over single-layer disease networks combined and discovered interesting disease associations, we are confident that the accuracy of our method will increase in time as more of the missing parts of each network are uncovered.

In contrast with the bottom-up “disease module” approach, which starts with the known genetic determinants of diseases to build disease modules and infer disease-disease interactions based on the localization of these modules in the underlying protein-protein interaction (PPI) network,^3,9^ we used a top-down approach, where the starting point was diseases themselves rather than the PPI, and recovered the underlying molecular mechanisms that associate with those diseases. One advantage of the top-down approach in characterizing diseases is that the construction of the disease-disease network relies on the unambiguous and relatively relaxed criterion of one shared gene or one shared symptom, making in easier to merge and parse large disease-related knowledge bases such as OMIM and Disease Ontology. Overall, it is worthwhile to compare and contrast the top-down and bottom-up approaches in future studies as they are likely to offer complementary benefits and possibly a significant overlap of results. For instance, one can envision a scenario where network-based similarity methods such as topological overlap can be used to calculate intra-community similarities to refine the disease groupings even further. Various network overlap metrics such as the separation metric^9^ can be used to determine disease similarity at the interactome level. We also recognize the possible limitations of our current “unweighted” network approach, where a link is established between diseases if the minimal criterion of one shared symptom or gene is met regardless of how many symptoms or genes are shared between them, such as the possible over-inclusion of edges due to common symptoms. However, our aim at this initial stage is to work on the unweighted network to serve as a minimal model of disease-disease interactions in the genotype- and phenotype-layers. While within the scope of this work we are able to validate the resulting multiplex disease groupings through intra-community similarity assessment and the recovery of known disease-disease relationships, we find it important to note that the results can be improved by treating each layer as weighted in future studies.

Our selection of diseases is geared toward maximizing the size of the dataset and is, therefore, biased towards neither complex nor Mendelian diseases. It, rather, includes diseases of a wide range of prevalences and penetrances, from rare congenital diseases to common cancer types and metabolic disorders. Our approach, however, seamlessly integrates the two types of diseases, with OMIM and GWAS evidence alike. For instance, dedicated and up-to-date GWAS datasets from the GWAS Catalog (https://www.ebi.ac.uk/gwas/) can be used in a similar fashion to build the genotype-based layer of alternative multiplex disease networks.

At the core of our disease classification is a technique that takes into account the information flow within and across different network layers. In the context of pathobiology, we use the term “information flow” to refer specifically to the process by which distant diseases can “communicate” by propagating genetic and symptomatic signals through intermediary diseases in the global disease network whereby, for example, disease A, which shares certain genes (or symptoms) with disease B, can be related indirectly to disease C, which also shares certain genes (or symptoms) with disease B. The diseases sending and receiving more information “flow” from each other in the genotype-layer would likely have related molecular roots, whereas in the phenotype-layer, this would mean groups of diseases that share clinical manifestations. We note that the use of quotes in “communication” is in order to emphasize that this is a process that does not involve actual molecular signaling but rather a conceptual one that implies associations between diseases. Capable of traversing both layers of disease networks, this method can put the same disease into two different communities based on both genotypic and phenotypic data. This “redundancy” of disease communities, with “bridge diseases” connecting them, is a key component of the next generation of organic, flexible disease classifications in which we can develop a network of disease communities rather than the current tree-like, hierarchical classification where a disease can only belong to one community. We believe that the common biology of these bridge diseases deserves further detailed investigation, and note it as an important future direction.

Overall, this study provides a rich compendium of disease associations and groupings that can be examined from many aspects ranging from relationships between complex and Mendelian disorders, to possible commonalities between communities of diseases. To our knowledge, this is the first time relationships between diseases have been assessed from the perspective of multiplex networks using novel network techniques designed specifically to uncover systemic properties at multiple levels. Furthermore, it offers evidence of potential molecular connections between diseases with similar clinical manifestations, given that there are many such diseases whose molecular connections to each other in the interactome are yet to be determined. While one can take each pair of phenotypically similar complex diseases and look into their molecular constituents to identify any molecular commonalities with a dedicated study, our approach offers a first step to do so globally.

A notable finding supported by clinical and genetic observations is that rare, Mendelian disorders predispose individuals to more common, complex diseases.^37^ In fact, in many of the disease communities, we observe monogenic disorders grouped together with the complex diseases for which they increase risk (See Table 1). For example, patients with Denys-Drash Syndrome, which is a rare disorder characterized by abnormal kidney function believed to be due to a mutation in the WT1 gene, have an estimated 90 percent chance of developing a rare form of kidney cancer known as Wilms tumor. Affected individuals may develop multiple tumors in one or both kidneys, testes, or ovaries. We note that this disease, along with Frasier syndrome, is grouped together with cancer types in disease community 16. Similarly, it has been documented that DiGeorge syndrome shows an increased risk of schizophrenia,^49^ which are both grouped in disease community 21. Disorders related to the homozygous or compound heterozygous deletions and loss-of-function mutations in NPHP1, such as Joubert Syndrome or Senior-Loken syndrome, have been linked to autism spectrum disorder,^50^ all of which can be found in disease community 12. Individuals affected by Wolfram syndrome have diabetes mellitus and degeneration of the optic nerve, and we note these diseases in community 22. Many more examples in line with the complex-Mendelian disorder associations can be appreciated by inspecting our disease communities. Our disease communities are, therefore, inclusive units wherein complex and Mendelian disorders are linked with both genetic and clinical factors.

**Table 1:** Example multiplex disease communities where monogenic disorders were grouped together with the complex diseases for which they increase risk (Communities 12, 16, 21, and 22), and Mendelian diseases with severe phenotypes were found in smaller communities (Communities 31, 40, 73, and 82)

Multiplex disease community #	Diseases
12	asphyxiating thoracic dystrophy, **autistic disorder**, Bardet-Biedl syndrome, erythropoietic protoporphyria, generalized epilepsy with febrile seizures plus, glycogen storage disease IV, hypermethioninemia, **Joubert syndrome**, Meckel syndrome, nephronophthisis, nephrotic syndrome, orofaciodigital syndrome, renal-hepatic-pancreatic dysplasia, **Senior-Loken syndrome**, thrombophilia, triple-A syndrome
16	**basal cell carcinoma**, **breast cancer**, Denys-Drash syndrome, **desmoplastic medulloblastoma**, Fanconi’s anemia, **Frasier syndrome**, **hereditary breast ovarian cancer**, holoprosencephaly, **malignant mesothelioma**, **medulloblastoma**, **nephroblastoma**, nephrotic syndrome, **nevoid basal cell carcinoma syndrome**, **pancreatic cancer**
21	alcohol dependence, cerebrovascular disease, **DiGeorge syndrome**, essential hypertension, factor V deficiency, factor XIII deficiency, Hermansky-Pudlak syndrome, homocystinuria, panic disorder, prothrombin deficiency, **schizophrenia**, thrombophilia
22	**cataract**, **diabetic ketoacidosis**, Donohue Syndrome, **hyperinsulinemic hypoglycemia**, **maturity-onset diabetes of the young**, nonpapillary renal cell carcinoma, pancreatic agenesis, renal cell carcinoma, **type 1 diabetes mellitus**, **type 2 diabetes mellitus**, **Wolfram syndrome**
31	achondrogenesis type IB, **atelosteogenesis**, Beare-Stevenson cutis gyrata syndrome, Boomerang dysplasia, diastrophic dysplasia, Larsen syndrome, multiple epiphyseal dysplasia, osteoarthritis, pseudoachondroplasia
40	cardiofaciocutaneous syndrome, Coffin-Lowry syndrome, cutaneous porphyria, fragile X syndrome, non-syndromic X-linked intellectual disability, Rett syndrome, **Smith-Lemli-Opitz syndrome**
73	bronchiectasis, Camurati-Engelmann disease, **cystic fibrosis**, Liddle syndrome
82	glycine encephalopathy, **Pfeiffer syndrome**, Rubinstein-Taybi syndrome

The diseases mentioned in the Discussion are highlighted in bold

Another interesting observation is that some Mendelian diseases with severe phenotypes, which were believed to be completely penetrant but were recently identified to be present in individuals with no apparent clinical manifestations,^51^ were found in our dataset within the smaller disease communities with size <10 (Cystic fibrosis: disease community 73(4), Smith-Lemli-Opitz syndrome: disease community 40 (7), Pfeiffer syndrome: disease community 82 (3), atelosteogenesis: disease community 31(9)) (See Table 1). This observation may point to the fact that these severe childhood diseases, which may include individuals resilient to them, tend to avoid being classified alongside other diseases, but, rather, have their own small cohesion groups. Although the findings of that particular study currently remain to be fully verified, it nevertheless represents an interesting research frontier wherein genetic modifiers that affect phenotypic variability are found through phenotype-genotype correlations across multiplex networks ultimately to identify “fully penetrant” Mendelian diseases that can be harbored by resilient individuals more clearly.

The results of our work can be refined and built upon in many ways. With the increased momentum towards precision medicine, efficient subtyping of diseases becomes a crucial need. To fill this need, multiplex disease networks that include a number of disease subtypes can be analyzed and re-grouped using network techniques. Similarly, a better classification of spectrum disorders, which are a collection of multiple diseases with some underlying commonalities, represents an important frontier of disease characterization. From that perspective, complex diseases that are regarded as spectrum disorders can benefit from the new associations and disease groupings that our approach provides. Another important direction would be the application of multiplex network-based approaches geared toward personalized diagnosis and treatment, with a focus on drug discovery and target identification.^52,53^ Finally, since the field of multiplex networks and the subsequent methods to detect communities within them is a very rapidly evolving one, more sophisticated multiplex community detection methods offer the possibility of further improving the characterization of diseases and identification of disease groups in the future. Indeed, the use of a host of newly emerging community detection methods tailored for multiplex networks^54–56^ presents an exciting future direction where they can be applied on global multiplex disease networks similar to ours to find further novel disease-disease associations and add many more layers of molecular information to disease classification.

## Methods

### The genotype data set

The disease-gene bipartite network is built from the well known Online Mendelian Inheritance in Man (OMIM) data set. OMIM (World Wide Web URL: http://omim.org/)^57,58^ is a knowledge base whose content is derived exclusively from the published biomedical literature describing human phenotypes and genes. The version used in this study contains information about 3369 genes and 4239 diseases, for a total of 5308 edges. The bipartite network of gene-disease interactions was projected onto the disease component to build the genotype-based disease-disease network layer.

### The phenotype data set

The disease-symptom bipartite network is built from the well known Human Phenotype Ontology data set. Human Phenotype Ontology^59^ provides a structured, comprehensive and well-defined set of 10,088 classes (terms) describing human phenotypic abnormalities and 13,326 subclass relations between the HPO classes. We have found 6662 diseases with relationships to 11,052 symptoms, for a total of 100,281 edges. The bipartite network of symptom-disease interactions was projected onto the disease component to build the phenotype-based disease-disease network layer.

### Linking genotype and phenotype data sets

Among the 3919 diseases common to both databases, we focused our attention on the subset of diseases matching the Disease Ontology database. Disease Ontology^60^ represents a comprehensive knowledge base of developmental and acquired human diseases. It semantically integrates disease and medical vocabularies through extensive cross mapping and integration of several disease-specific terms and different identifiers. We found 2255 matching unique OMIM identifiers for 910 diseases. Finally, we discarded the isolated diseases without any links in either layer, which resulted in 779 diseases in the multiplex network. Despite the seemingly high number of diseases in the OMIM and HPO databases, a large number of these disease names are synonyms of each other, resulting in a much smaller number of diseases when semantically integrated through the Disease Ontology database. While integrating databases inevitably results in the exclusion of some diseases, we ensured that our multiplex disease network, which has close to 800 diseases, represents the largest available dataset satisfying our strict criteria. The resulting multiplex diseasome is publicly available at the following URL: https://github.com/manlius/MultiplexDiseasome, and data and an interactive web applet for the exploration of multiplex disease communities is available at https://github.com/r-duh/MultiplexDiseasomeCommunities.

### Significance of overlapping interactions across layers

We find that 139 edges overlap between the two layers (Fig. S1), significantly higher than what would be expected at random (31.80 ± 5.15 edges). The randomization scheme is such that we keep the degree distribution of each network fixed while randomizing the edges per layer for a total number of 5000 times. The distribution resulting from this null model is normal (Shapiro–Wilk test *P* = 0.33) and the *z*-score for the observed overlap is 20.8 (Fig. S2).

### Gene similarity of disease pairs

An important indicator of whether or not diseases connected by genotypic or phenotypic relations have a substantially similar genetic background is the gene overlap of disease pairs. To quantify this gene overlap, we calculated the Jaccard index *J* of the gene sets of diseases connected by an edge (SI Section 13). We then calculated the average Jaccard index 〈*J*〉 overall edges in the genotype layer, the phenotype layer, and the edge overlap network, i.e., the network obtained from the multiplex disease network by considering only overlapping disease-disease interactions. We compared the average Jaccard indices of each network with the random expectation, calculated by generating ensembles of networks with the same degree distribution in each layer separately. We observed that the gene similarity of disease pairs in the phenotype network is comparable to random expectation due to the non-specificity of many symptoms, whereas for the genotype layer and the edge overlap network, the gene similarity is significantly different from random expectation (Fig. S25). Moreover, the gene similarity of the overlap network is higher than both the genotype and phenotype layer. This suggests that the additional layer of information provided by the phenotype layer, despite having little gene overlap overall itself, helps filter out the disease pairs in the genotype layer that have higher gene overlap, reinforcing the genetic effect between disease pairs. In other words, arising as a natural feature of the data, disease pairs whose relationship is supported by both genotype and phenotype evidence have a higher gene overlap than the genotype layer, which hints at the complementary and cooperative nature of these two factors worthy of further investigation.

### Community detection on single and multilayer networks

Infomap is an algorithm to optimize the map equation.^38,39^ This information-theoretic equation makes extensive use of the duality between the problem of compressing a data set and the problem of revealing significant structures within it. To achieve its goal, the algorithm makes use of random walkers to explore the network, encoding their flow with sequences of bits. In our case, the data set is a network and the (unknown) significant structures are the communities. Each possible community partition of the network result in a specific encoded flow: the partition whose description length is minimum is the optimal one. Therefore, the Infomap algorithm exploits the dynamics on the network to reveal the underlying community structure.

The generalization of this algorithm to the case of multilayer networks, called Multiplex Infomap,^32^ is based on the same principle. In this case, the random walkers explore all of the layers of the multilayer structure within which there is a parameter, the relax rate, regulating the probability of visiting more nodes within the same layer than across layers. In other words, this parameter is used to modulate the “flexibility” of movement across layers in the absence of information on the actual interlayer link weights. Numerical experiments^32^ show that values of the relax rate close to approximately 0.5 are generally good enough to balance exploration within and across layers. We accordingly use a relax rate of 0.45 throughout the analysis (see SI Section 3 and Fig. S6 for a sensitivity analysis for determining the relax rate).

### Similarity measures: MimMiner

In order to determine the phenotypic similarity of the diseases in a given cluster, we used the MimMiner similarity matrix.^5^ MimMiner assesses the semantic similarity of phenotypic terms related to diseases in the OMIM database. For each OMIM disease, it builds a feature vector consisting of Medical Subject Headings (MeSH) concepts that collect all synonyms and uniquely identify terms, which makes it a more versatile method than keyword–based searches. The MimMiner similarity score is calculated using these feature vectors, resulting in normalized values between 0 and 1. In our analyses, we used the MimMiner similarity matrix to determine the intra-community similarity of all of the 30 communities of diseases. Of the 779 OMIM diseases we consider in our dataset, 675 (87%) were mapped to the MimMiner matrix. We follow the cutoff of 0.3 proposed in the original paper^5^ to define associations that are biologically informative, whereas we deem scores above 0.6 to be significantly functionally similar.

### Similarity measures: gene ontology based on gene set similarity

For an insight into the similarity of the molecular mechanisms underlying the diseases in our disease communities, we make use of the GO–based gene set similarity measure proposed in.^61^ We prefer the gene set based similarity over pairwise gene similarity measures since pairwise gene similarity does not scale as well to large gene sets and since we aim to compare pairs of diseases in each community, many of which have multiple genetic elements. This measure essentially lets us rank each GO term with respect to a gene set based on the number of genes in the set that are annotated by the ancestors of that GO term. We limit our attention to Biological Process. Furthermore, for concreteness, we only consider evidence codes EXP, IPI, IDA, IMP, IGI, IEP, ISS, ISA, ISM, and ISO, which are either experimental or computational analysis evidence codes.

### Similarity measures: comorbidity (relative risk)

Another similarity measure we use in assessing the homogeneity of our disease communities is comorbidity, i.e., the co-occurrence of diseases in the same patient. For this, we use a healthcare dataset comprising the patient history of 13 million elderly Americans over the age of 65 covered by the Medicare program.^6^ We manually curate our disease set consisting of 779 diseases to reflect the 3-digit ICD-9 disease classification code. We quantify the comorbidity of disease pairs using relative risk (RR) score, which is given by the ratio of the observed co-occurrence of a disease to the expected probability of co-occurrence if the diseases were independent from each other,

1 $$RR_{AB} = \frac{{C_{AB}/N}}{{P_AP_B/N^2}}$$

where *C*_*AB*_ is the observed co-occurrence of disease A and B, *P*_*A*_ and *P*_*B*_ are the prevalence of disease A and B, and *N* is the total number of patients in the dataset’s population. A relative risk greater than 1 indicates a comorbidity that is higher than expected. Since RR values can vary several orders of magnitude depending on the prevalence of a disease, we take the logarithm of RR values when visually representing them.

### Similarity measures: gene overlap

As an additional proxy of the molecular intra-similarity of disease communities, we calculate the direct gene overlap between pairs of diseases. This is intended to provide a direct and straightforward measure of shared genetic constituents. We then use Jaccard index J to quantify the gene overlap with

2 $$J_{AB} = \frac{{g_A \cap g_B}}{{g_A \cup g_B}}$$

where *g*_*A*_ and *g*_*B*_ are the gene sets of disease A and disease B.

### Publication co-occurrence

To validate the biological meaningfulness of the content of our disease communities, we seek to gain an overview of the literature associations of diseases from PubMed records. PubAtlas (World Wide Web URL: http://www.pubatlas.org/) is a web service that acts as a front end to the PubMed/MEDLINE database. Using PubAtlas, we generate “literature heatmap” for each disease community by querying the names of diseases within each community. The color in these heatmaps is given by the logarithm of the Jaccard association value. We also represent this heatmap with a network of literature associations where the color (same as the heatmap) and the width of the link reflect the strength of association. Using PubAtlas hence lets us identify the disease pairs in a disease community that have known former associations through literature, providing us with a basis for validating the community as well as distilling its basic characteristics.

## Supplementary information

Supplementary Information and Tables

## Data Availability

The data that support the findings of this study are available from the corresponding author upon reasonable request.

## Supplementary information

**Supplementary information** accompanies the paper on the *npj Systems Biology and Applications* website (10.1038/s41540-019-0092-5).
