# Supplementary material for: The multiplex network of human diseases
Source: NPJ Syst Biol Appl. 2019 Apr 23;5:15. doi: 10.1038/s41540-019-0092-5 (PMC6478736; doi:10.1038/s41540-019-0092-5)
Supplement: Supplementary file 1 — Supplementary Information and Tables [file 41540_2019_92_MOESM1_ESM.pdf]

The multiplex network of human diseases
Arda Halu, Manlio De Domenico, Alex Arenas, Amitabh Sharma

Supplementary Information

1 Edge overlap between genotype and phenotype layer

To assess the edge overlap, we compare the number of observed overlapping links (139 links overall, Fig. S1) to what would be expected by random. To randomize the network, we keep the degree distribution of each layer fixed while randomizing the edges of each layer. We repeat the randomization process 5000 times and record the number of edge overlaps in each case, resulting in a distribution of overlaps in the randomized networks to serve as a null model. We verify the normality of this distribution through the Shapiro-Wilk test (p-value = 0.33) and then calculate the z-score of the observed overlap, which is 20.8 (Fig. S2).

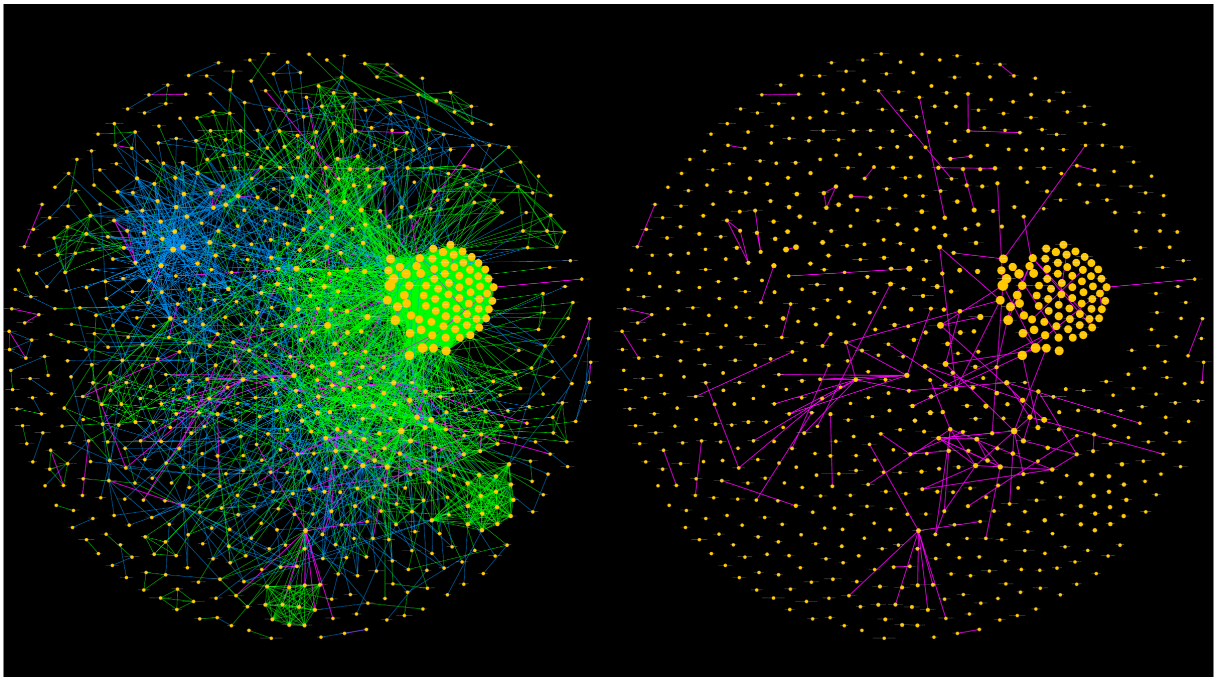

Figure S1: (Left) Multiplex disease network with both layers superimposed. Nodes represent diseases and green and blue links represent disease relationships in the phenotype-based and genotype-based layer, respectively. Overlapping links are marked by magenta links. (Right) Multiplex disease network with only the overlapping links shown for clarity.

2 Structural organization of the two layers

Given that the two layers have very different edge densities (0.0074 and 0.0253 for genotype and phenotype, respectively), we want to further examine the different topological features, especially the different granularities of the two layers. We first plot their degree distribution and

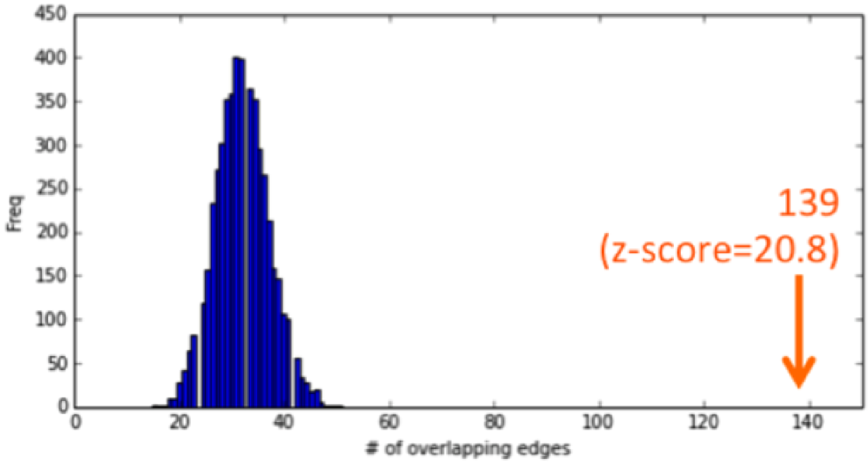

Figure S2: The observed overlap is statistically highly significant against a background of randomized network realizations.

the clustering coefficient distribution. We find that while both layers have heavy tailed degree distributions, the maximum degree in the phenotype layer is much higher than the genotype layer (Fig. S3). This is due to the criterion for forming a link between diseases for the phenotype layer (i.e. if they share a symptom) than the respective criterion for the genotype layer (i.e. if they share a disease gene), following from the fact that many symptoms are rather non-specific, resulting in potentially many connections for a given disease.

Another important aspect underlying the structural difference between the two layers is the distinct levels of heterogeneity of the two networks. In order to study this aspect and learn more about the local granularity of each network, we plot their local clustering coefficient distribution $P(C_i)$. We find that the distribution of local clustering coefficients is bimodal in both cases, owing to the presence of both densely clustered regions with C_i close to 1 (due to diseases with wide-ranging symptoms, such as multi-system diseases, in the phenotype layer; and complex diseases involving numerous gene mutations, such as cancers, in the genotype layer) and sparsely connected regions C_i close to 0 in both networks. However, the two networks are significantly different ($P = 5.25E - 13$, two-sided Mann-Whitney U test) from each other in which type (C_i close to 0 or C_i close to 1) is more predominant in the network (Fig. S4. In particular, we note that the phenotype layer has more of the clique-like group of diseases (also the root of its bimodal degree distribution), which results in a higher portion of nodes with $C_i = 1.0$. The opposite is true for the genotype layer where we see a higher portion of nodes with $C_i = 0$, indicating that, overall, the clustering is weaker compared to the phenotype layer.

Following from the presence of heterogeneous dense regions in each layer, we observe that the overlapping links tend to be localized around what can be called "overlap hubs" rather than placed homogeneously. Indeed, the heavy-tailed degree distribution of the overlap links confirms this observation (Fig. S5). A closer look at the diseases that have a high number of edges in the overlap network reveals that these are either groups of diseases (such as retinis pigmentosa, $k = 8$) or multi-system diseases (such as lamb-girdle muscular dystrophy, $k = 8$) that affect a number of organs and have wide ranging symptoms as well as common genetic factors with other diseases, resulting in their hub status in the overlap network.

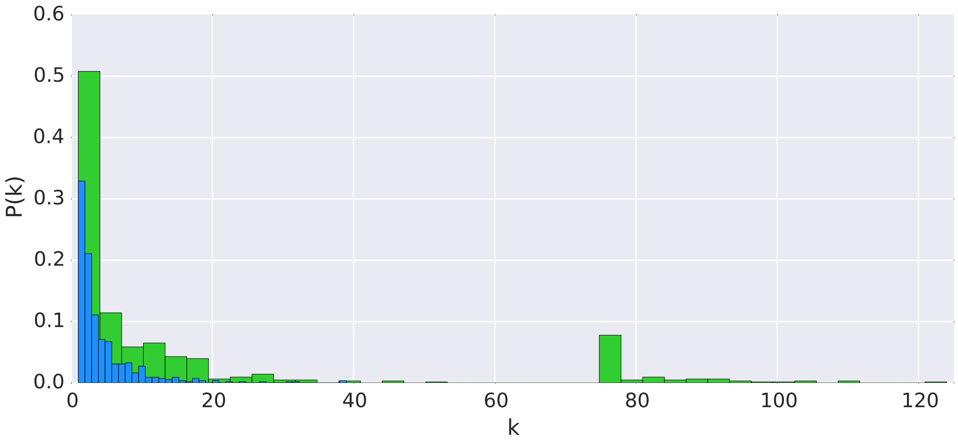

Figure S3: The degree distribution $P(K)$ of the genotype (blue) and phenotype (green) layer.

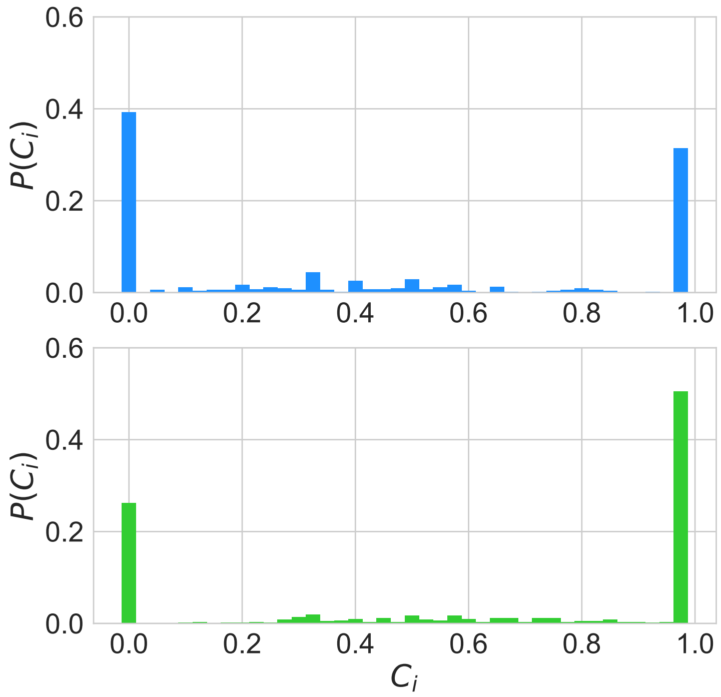

Figure S4: The local clustering coefficient distribution $P(C_i)$ of the genotype (blue) and phenotype (green) layer. The distributions are statistically significant (P=5.25e-13 by two-sided Mann-Whitney U test).

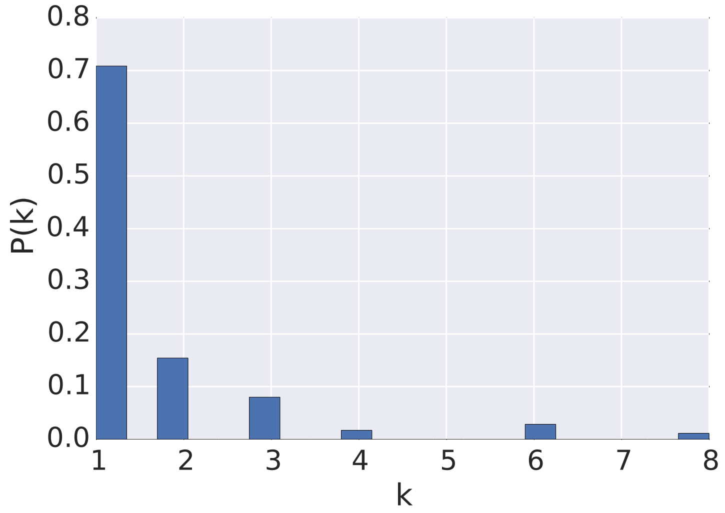

Figure S5: The degree distribution $P(K)$ of the overlap network.

3 Notes on the use of multiplex Infomap and the choice of relax rate

In the case of single-layer networks, different community detection methods provide, in general, different results, unless the community structure is extremely strong. However, while in the single-layer case there are many algorithms that can be used and compared, for instance to obtain an overall consensus, in the case of multiplex networks this is not possible. There are only a couple of grounded methods, attempting to generalize single-layer approaches. One of them is the multislice modularity maximization introduced by Mucha et al [1], which depends on a resolution parameter and on the weight (uniformly) assigned to inter-layer connections. The main drawback of this method is that in our case inter-layer links are missing and not even theoretically reasonable to explain. Instead, the multiplex version of the InfoMap algorithm has been designed *ad hoc* to cope with the absence of inter-layer links and depends only on one parameter, the relax rate. The relax rate might seem arbitrary, therefore, in absence of further information, we invoke the maximum-entropy principle and choose the relax rate that equally gives importance to local exploration and global teleportation, i.e. $r = 0.45$. To demonstrate the robustness of the results with respect to changes in the relax parameter, we compare the results we obtained from the data against an ensemble of $M = 199$ Monte Carlo realizations representing a specific null model. In our null model, the degree distribution of each layer is preserved but intra-layer correlations are destroyed by rewiring the links, whereas inter-layer correlations are preserved, because each node keeps the original number of links (ie, its degree) in each layer separately. We therefore apply our community detection algorithm to the ensemble and perform a non-parametric test to quantify the significance of our findings. We use the Wilcoxon–Mann–Whitney test, where the value $s^\star$ of the statistics obtained from the data (in this case the code-length and the number of modules) is compared against the values s_i $(i = 1, 2, ..., M)$ obtained from the random realizations of the null model. The two-sided test rejects the null hypothesis that the data are compatible with the null model either if $s^\star < s_i$ or $s^\star > s_i$ for all i. The statistical significance of the test is $\alpha = 2/(M + 1)$, and in our case it is 1%. Figure S6 shows that our statistical test rejects the null hypothesis either using code-length (top panels) or number of modules (bottom panels) as test statistics, regardless of the relax rate used (here

we show the results for $r = 0.45, 0.50$ and 0.55). We can therefore assess that the clusters of diseases found by our algorithm are significant with 99% confidence level with respect to a null model where the networks in the two layers preserve the underlying degree distribution and inter-layer correlations. The significance of the result is further reduced if inter-layer correlations are destroyed as well, by randomly relabeling the nodes.

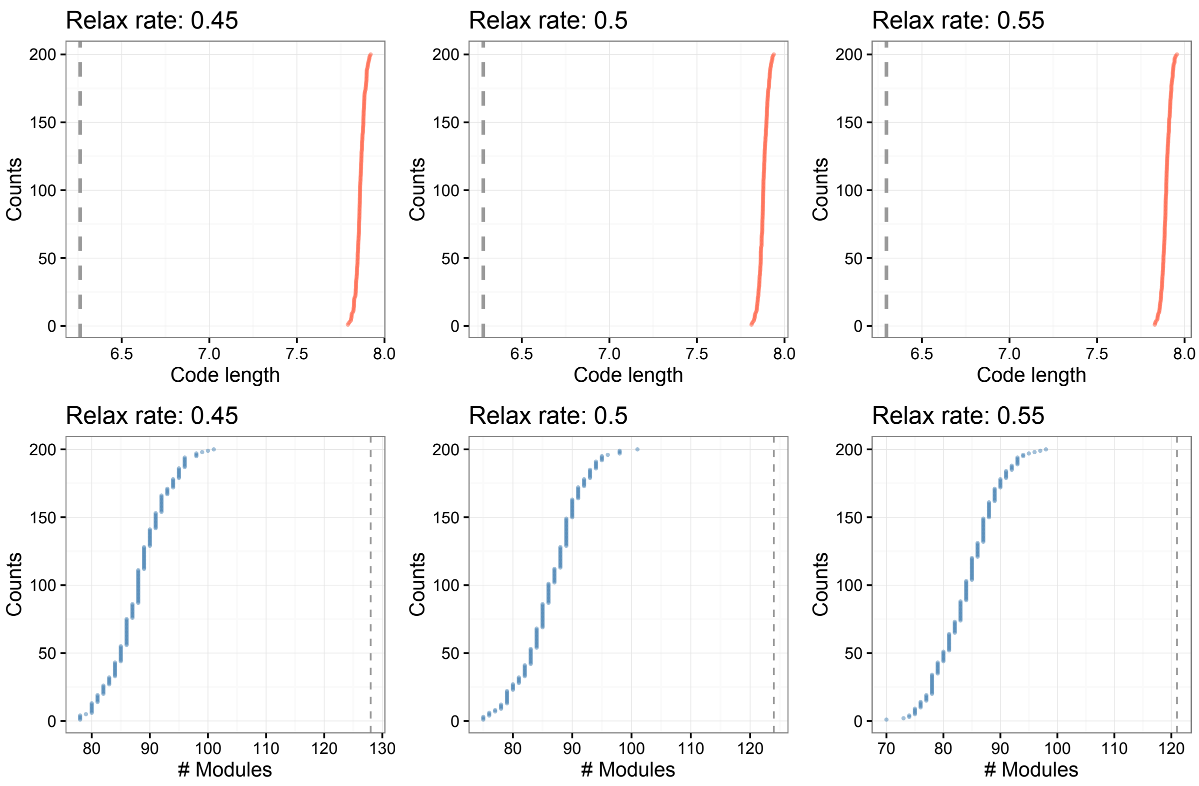

Figure S6: The Wilcoxon–Mann–Whitney statistical test rejects the null hypothesis either using code-length (top panels) or number of modules (bottom panels) as test statistics, regardless of the relax rate used (here we show the results for $r = 0.45, 0.5$ and 0.55). The dashed lines indicate the respective test statistic $s^{\star}$ obtained from data whereas the data points indicate the text statistics s_i (red for code length and blue for number of modules) obtained from the random realizations.

4 Specificity of single-layer disease communities

We first detect communities for the genotype and the phenotype layer separately using the Infomap algorithm. Then, to find out whether there is significant disease overlap of communities found separately in each layer, we calculate the average Jaccard index for the disease overlap between all possible genotype-phenotype community pairings. This gives us an observed average Jaccard index $\langle J \rangle = 0.00286$. To check if this indeed indicates a low overlap, we then follow the same procedure on randomized ensembles for a total number of 5000 realizations. Since the topological aspects of each network are crucial in the resulting community structure, we randomize the network in each layer by keeping the degree distribution constant, using degree-preserving randomization. The mean of the average Jaccard distribution of this randomized

ensemble is $\langle J \rangle = 0.00261 \pm 0.000289$, resulting in a picture where the actual disease overlap is indistinguishable from the random case with a z-score = 0.882 (Fig. S7).

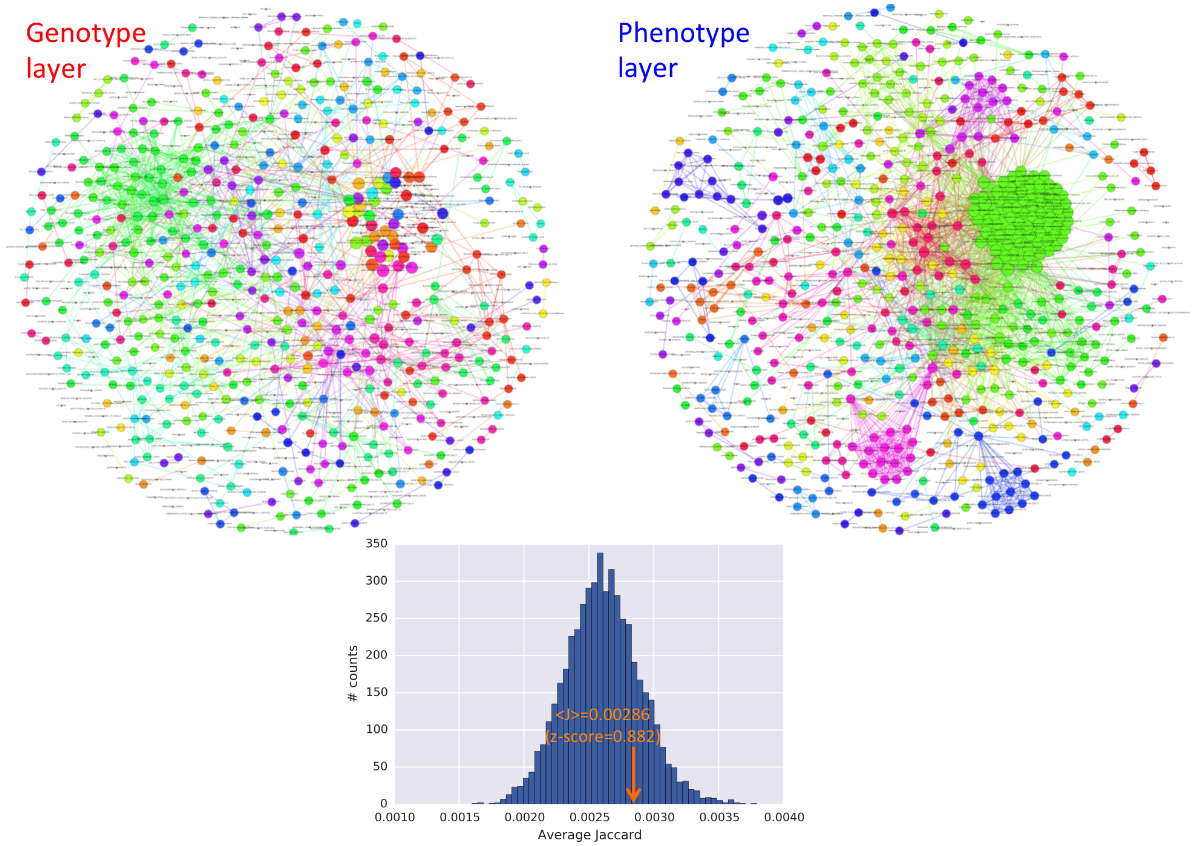

Figure S7: The communities found by the Infomap algorithm separately on the two layers have a low overlap comparable to random expectation.

5 Size distribution of multiplex disease communities

Overall the 779 diseases are divided into 128 multiplex communities. Out of the 128 disease groups, the largest one has 91 diseases, whereas the smallest one has 2 diseases. The number of unique diseases classified into groups based on Multiplex Infomap reaches a plateau rapidly, with the disease communities of size greater than or equal to 10 comprising 520 diseases (Fig. S8).

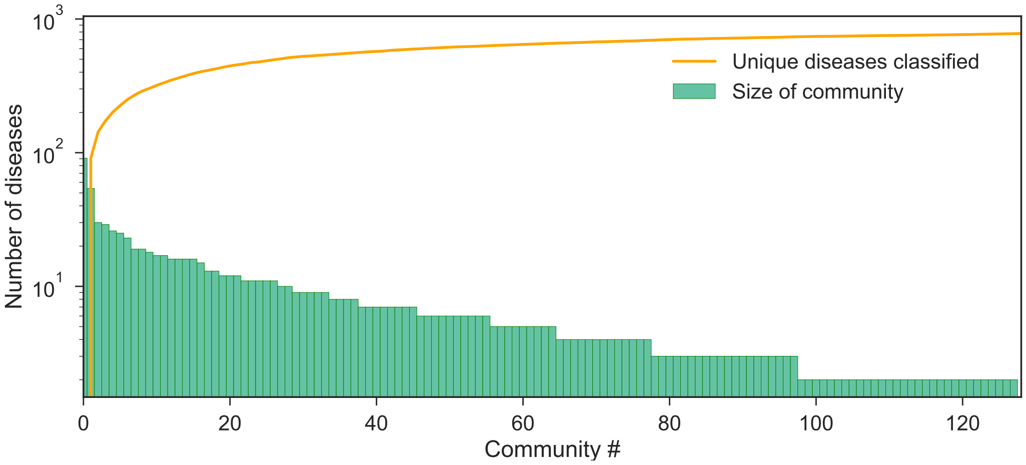

Figure S8: The size distribution of disease communities and the cumulative number of diseases classified into groups, as a function of disease communities, sorted from largest to smallest.

6 Bridge diseases

As a result of the multiplex community detection method, many diseases are classified into two different communities, acting as "bridges" between the two communities that they belong to. Among 779 diseases, 215 are in this category. For the 29 largest disease communities that have a size greater than 10, the connections between communities are shown in Fig. S9. We note that some smaller disease communities are more abundant in terms of the number of bridge diseases they have, such as disease community 23, 26 and 28. We compare the 215 bridge diseases with the remaining 564 non-bridge diseases to find out whether there are any discerning topological properties of bridge diseases. In particular, we compare the degree (sum of the number of connections, clustering coefficient, centrality, and strength (sum of weights) distributions of the bridge and non-bridge diseases. Our results show mostly no statistically significant difference between the two groups of diseases. For degree distributions, the Mann-Whitney U p-values are 0.40 and 0.11 for the genotype and phenotype layer, respectively. For clustering coefficients, the Mann-Whitney U p-values are 0.03 and 0.44 for the genotype and phenotype layer, respectively. The mean clustering coefficient of bridge and non-bridge diseases in the genotype layer are 0.48 and 0.41, respectively, which means that the bridge diseases have significantly higher clustering coefficient in the genotype layer than the non-bridge diseases. For eigenvector centrality, the bridge diseases have significantly lower eigenvector centrality than non-bridge diseases in both the genotype layer (0.012 vs 0.014, Mann-Whitney p-value 0.04) and the phenotype layer (0.0150 vs 0.0152, Mann-Whitney p-value 0.004). For betweenness centrality, the bridge diseases have significantly higher eigenvector centrality than non-bridge diseases in the phenotype layer (0.0022 vs 0.0017, Mann-Whitney p-value 0.006) but not the the genotype layer (0.0027 vs 0.0039, Mann-Whitney p-value 0.14). Finally, for strength distributions, the difference is not significant for either the genotype or the phenotype layer (with Mann-Whitney U p-values 0.38 and 0.07, respectively). These results are summarized in Fig. S10.

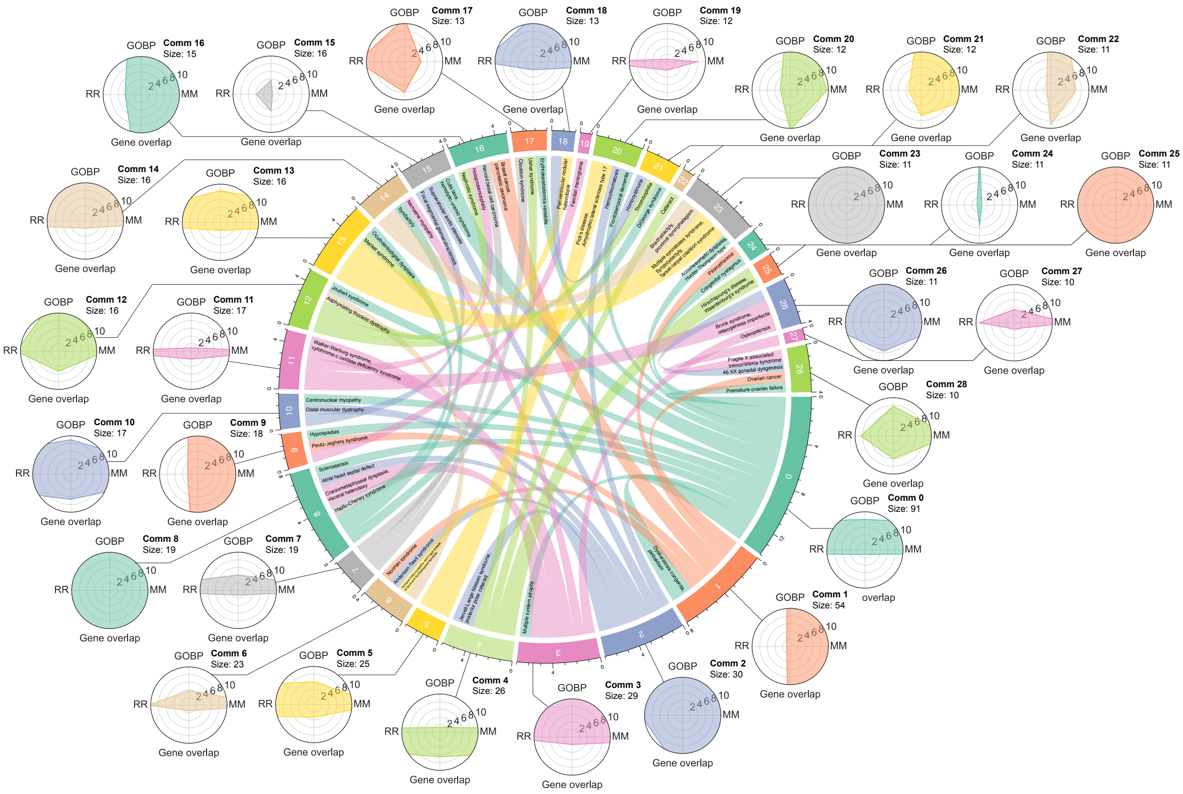

Figure S9: The connections between the largest 29 disease communities which arise from the bridge diseases common to both communities. The number within each band represents the disease community number, and the tickmarks outside the spheres count the number of bridge diseases shared. Diseases that bridge multiplex disease communities are labeled within the respective chord. Radar plots from Figure 3 depicting the molecular and phenotypic similarities of diseases contained within communities are shown next to their respective communities.

	Mann-Whitney U p-values				
	**Degree**	**Clustering coefficient**	**Betweenness centrality**	**Eigenvector centrality**	**Strength**
**Genotype layer**	0.40	0.03	0.14	0.04	0.38
**Phenotype layer**	0.11	0.44	0.006	0.004	0.07

Significantly higher in bridge diseases than non-bridge diseases
Significantly lower in bridge diseases than non-bridge diseases
Not significant

Figure S10: The comparison of bridge and non-bridge diseases in terms of their distributions of various topological measures.

7 Pairwise disease similarity heatmaps

To assess the intra-similarity of multiplex disease communities, we calculate the pairwise similarity of all disease pairs in the communities to use as the background distribution when calculating the significance of intra-similarity. The whole similarity matrices for GO: Biological Process, comorbidity as measured by relative risk, gene overlap Jaccard index, and semantic similarity are shown in Figs. S11, S12, S13, S14, respectively.

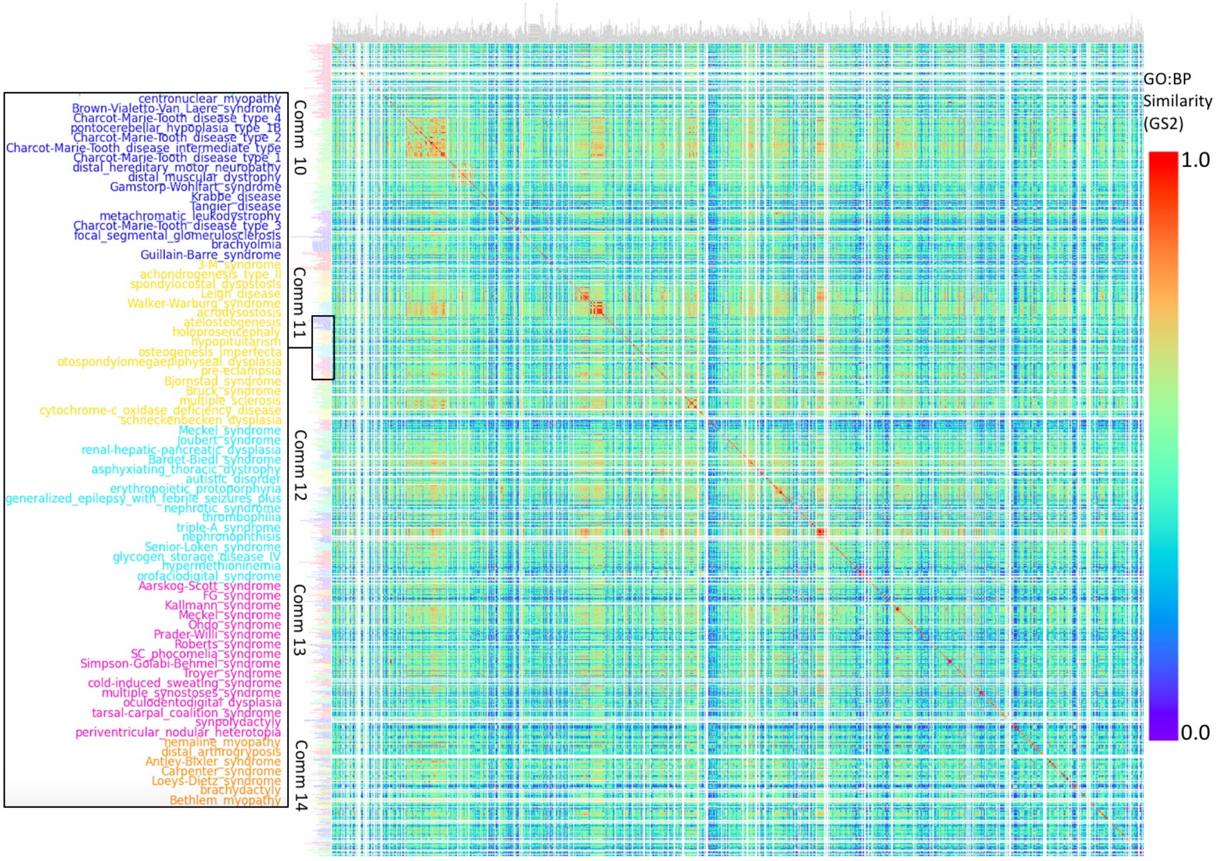

Figure S11: The pairwise GO: Biological Process similarity matrix of all diseases in multiplex disease communities, color coded, ordered in decreasing size.

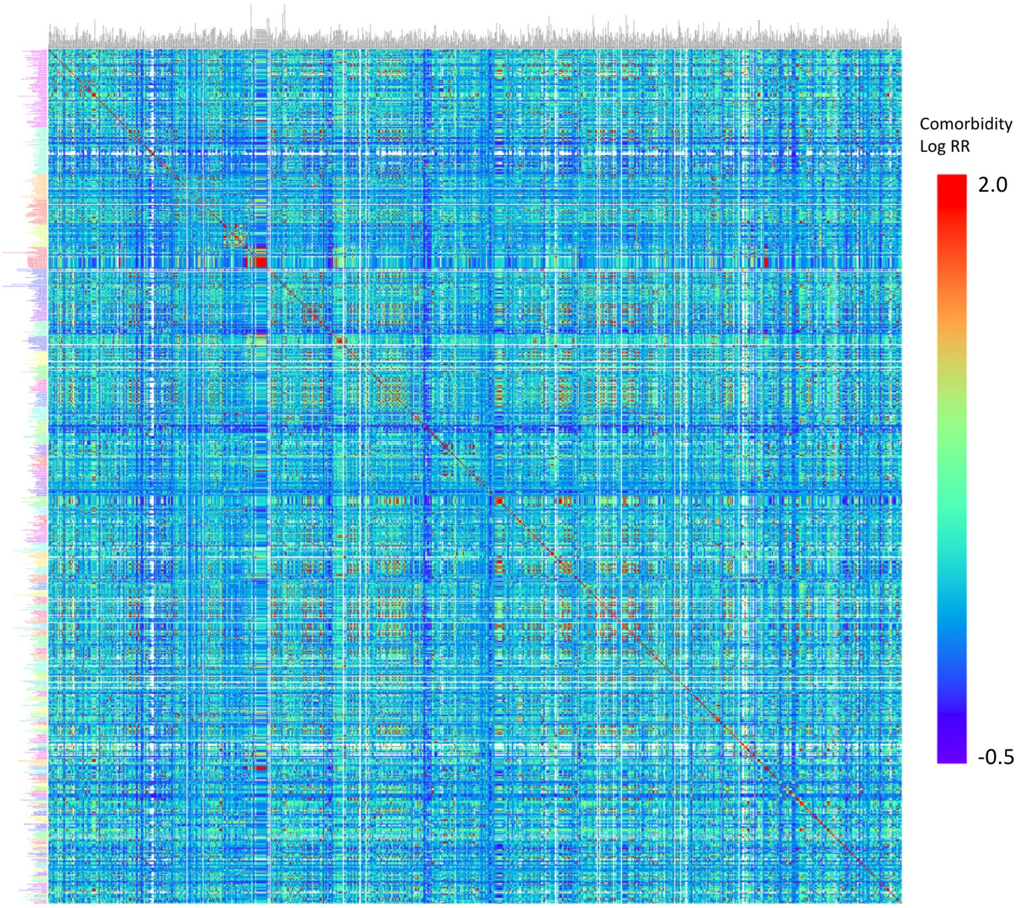

Figure S12: The pairwise relative risk comorbidity matrix of all diseases in multiplex disease communities, color coded, ordered in decreasing size.

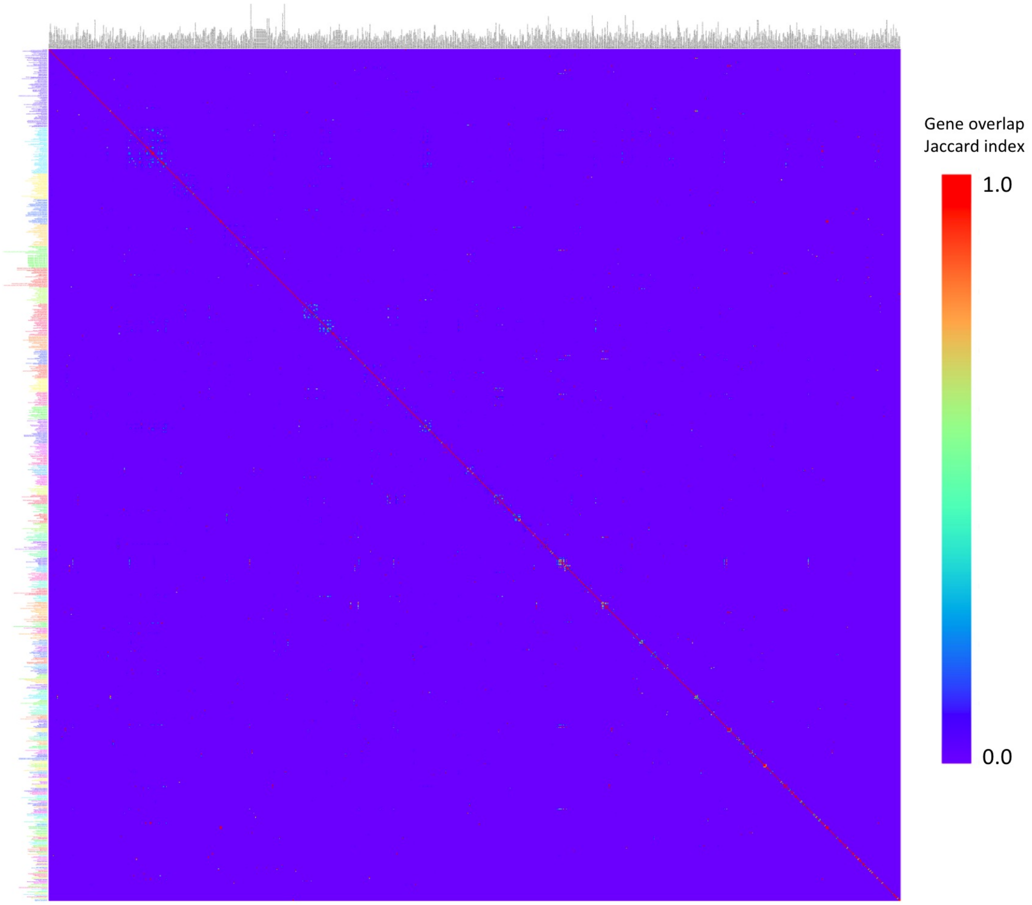

Figure S13: The pairwise gene overlap Jaccard index matrix of all diseases in multiplex disease communities, color coded, ordered in decreasing size.

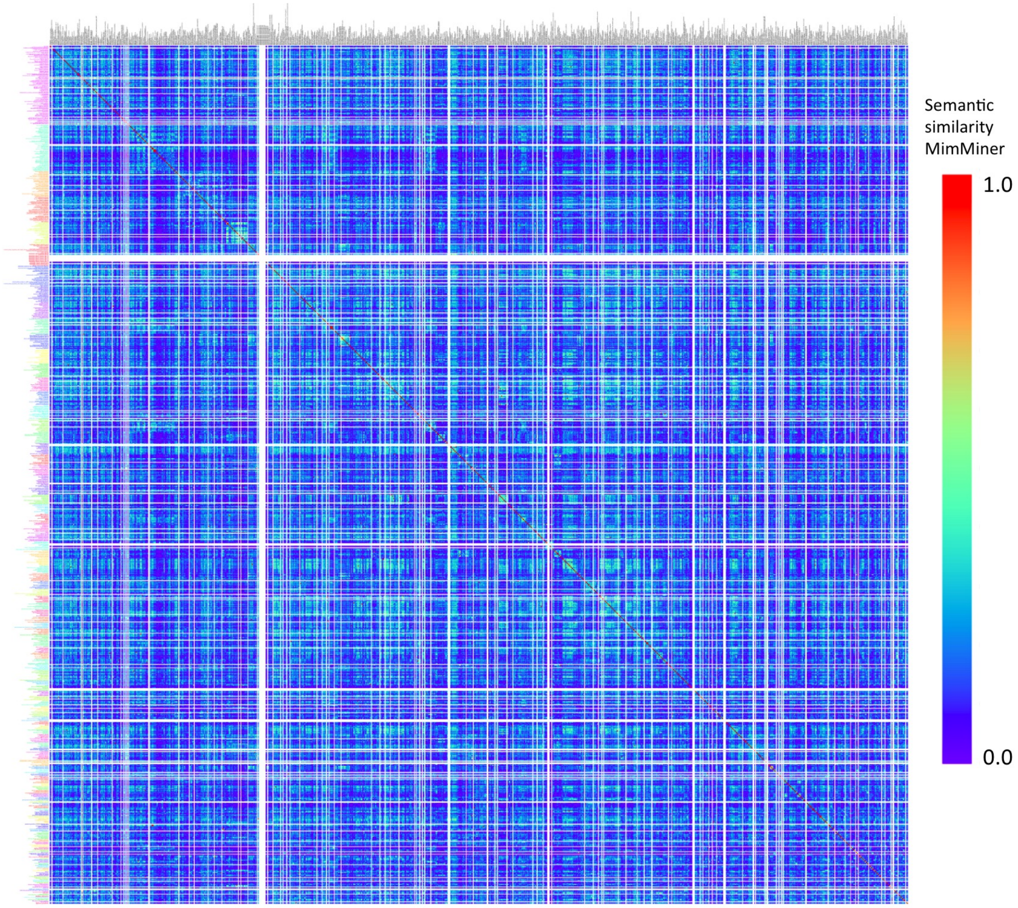

Figure S14: The pairwise MimMiner semantic similarity matrix of all diseases in multiplex disease communities, color coded, ordered in decreasing size.

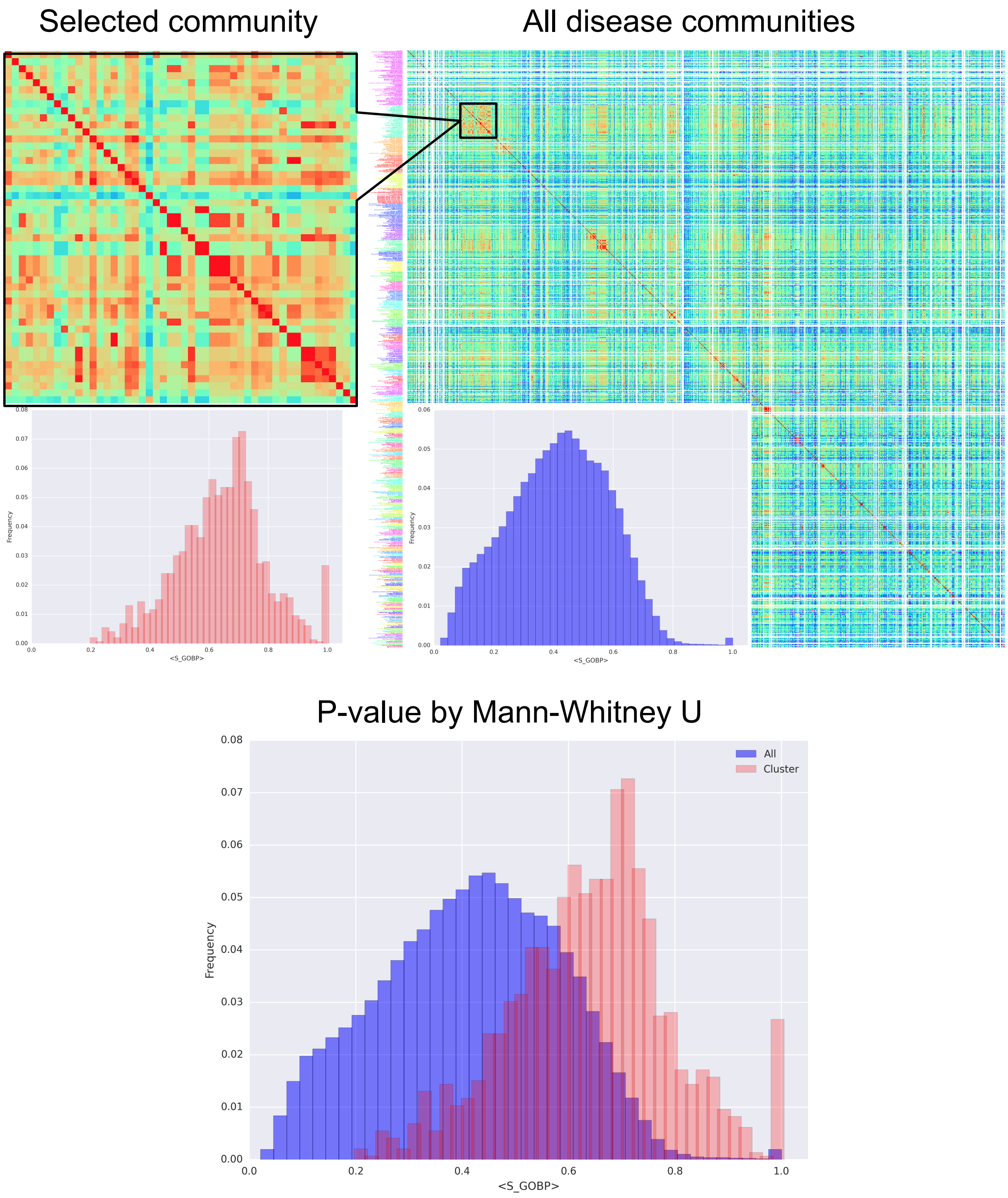

Figure S15: **Similarity assessment of multiplex disease communities.** Each disease community is evaluated for significance of intra-community similarity according to the four similarity measures. For each similarity measure, the distribution of similarity values for the disease community is compared against the background of all disease communities and its respective P-value is calculated by two-sided Mann-Whitney U test. Communities that have an average similarity higher than the background and a P-value smaller than 0.01 are considered as significantly similar for the given similarity measure.

8 Validation of potential disease associations using Disease-Connect

As an unbiased, global, quantitative comparison of all multiplex and individual layer disease communities, we next measured the disease communities' propensity to capture disease associations missing from both the genotype and the phenotype layers, thereby highlighting potentially novel disease associations that can be derived from our data that might be of interest to clinicians and biomedical researchers. We consider all multiplex and single-layer disease communities (Figure S16a), and construct the complete graph containing all possible edges between community diseases (Figure S16b). We then subtract the edges already in the genotype and phenotype layers, which leaves us with diseases that do not have a shared gene or symptom, but are potentially associated with each other (Figure S16c and S16d). To evaluate these potential associations, we turn to an independent and comprehensive disease association database (Disease-Connect), which assigns "strengths" to edges based on multiple sources of molecular evidence [2]. We measure the overlap of potentially novel edges within our multiplex communities with the edges that are captured by Disease-Connect (Figure S16e). As such, this validation analysis is simply a cross-check with an external "ground truth" rather than a direct inference of unknown edges, which is a separate domain of research outside the scope of this study.

Following this procedure, we found that in terms of the number of potentially novel edges evidenced by Disease-Connect, multiplex disease communities far outperform genotype and phenotype communities, with overall 146 overlapping edges for multiplex communities versus 61 and 15 for genotype and phenotype communities, respectively (Figure S16f). This result demonstrates that computing communities in the multiplex allows the uncovering of novel or underappreciated molecular disease associations, owing to the bridging of different communities that are separately represented in single-layers. We also measured the strength of the potentially novel edges in terms of the negative log of their association p-values. To adjust for the confounding factor that the number of diseases and, therefore, the possible edges between them may be higher for multiplex communities than single-layer communities, we calculated the mean -log(p-value) of all new edges for each case. Once again, multiplex communities have a higher mean disease association strength compared to both phenotype- and genotype-layer communities (Figure S16g). This result suggests that, even after normalizing for the number of edges where multiplex communities have an advantage, the mean association strength is higher for the multiplex communities than for both of the single-layer communities.

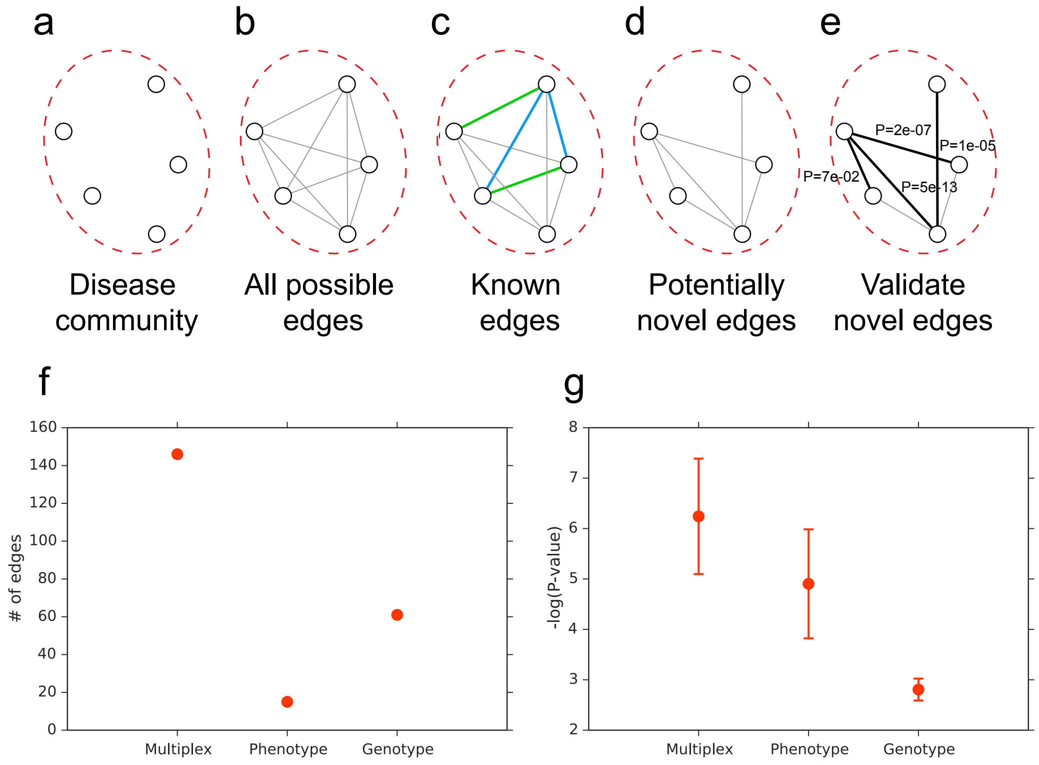

Figure S16: **Validation of potential disease associations.** **(a)** The disease list of a given multiplex or single-layer disease community is obtained. **(b)** All possible edges between the diseases in the disease community are created to represent all possible disease associations in the community. **(c)** The edges already in the genotype (blue) and phenotype (green) layers are determined. These edges represent the disease associations that are already known. **(d)** The known edges are subtracted from the possible edges. The remaining edges are the potentially novel disease associations within the disease community. **(e)** The possibly novel edges are validated: The overlap with the disease pairs in the Disease-Connect database and the significance of the overlapping edges are measured. **(f)** The number of overlapping genes with the molecular based disease associations in the Disease-Connect database. **(g)** The mean strength of disease associations for the overlapping edges in the Disease-Connect database. Error bars indicate standard deviation.

9 Multiplex disease communities: Comparison with randomized communities

In order to establish that the multiplex disease communities we found are biologically distinct from random communities, we randomize the pairwise similarity matrices by keeping the size of communities fixed, and compare the four similarity measures between the actual and the randomized case. Once again, we compare the similarity distribution of the each community with the background of all disease pairs using two-sided Mann-Whitney U test. We find that, within the top 30 communities with the largest size, there are no communities with mean RR comorbidity less than that of the background (i.e. of all diseases), and only three communities are not significant, whereas for the randomized case there are two communities with mean RR comorbidity smaller than background, and nine are not significant. Furthermore, the intra-community similarity –log(p-values) for RR comorbidity are significantly higher than those of randomized communities (mean(-logP): 30.72 vs. 6.54, $p = 0.00034$ two-sided Whitney-Mann U test). Similarly fo MimMiner semantic similarity, there are no communities with mean MimMiner similarity less than that of the background and only two communities are not significant, whereas for the randomized case there are seven communities with mean MimMiner similarity smaller than background, and ten are not significant. The intra-community similarity –log(p-values) for MimMiner similarity are significantly higher than those of randomized communities (mean(-logP): 33.40 vs 3.73, $p = 1.008E-08$, two-sided Whitney-Mann U test). For Gene Ontology: Biological Process (GO:BP) similarity, there are six communities with mean GO:BP similarity less than that of the background and only three communities are not significant, whereas for the randomized case there are four communities with mean GO:BP similarity smaller than background, and ten communities are not significant. The intra-community similarity –log(p-values) for GO:BP similarity are significantly higher than those of randomized communities (mean(-logP): 28.85 vs 5.41, $p = 0.00024$, two-sided Whitney-Mann U test). The reason that multiplex communities perform relatively closer to random for GO:BP similarity compared to other similarity measures is that the overall similarity of background is relatively higher for this measure, which could be due to many common endophenotypes and pathobiological pathways that loosely connect many diseases that are in different disease communities. Finally, for gene overlap (Jaccard index), there are once again no communities with mean gene overlap less than that of the background and only two communities are not significant, whereas for the randomized case, while there are no communities with mean gene overlap smaller than background, eighteen communities are not significant. The intra-community similarity –log(p-values) for gene overlap are significantly higher than those of randomized communities (mean(-logP): 10.10 vs 1.37 , $p = 5.98E - 09$. two-sided Whitney-Mann U test).

10 Biological cohesiveness of single-layer disease communities – a comparison with multiplex communities

It is crucial to assess how well the single-layer disease communities perform in terms of the four similarity measures, in order to be able to comment on the level of cohesiveness of the multiplex disease communities. To do this, we repeat the intra-community similarity measurements for the genotype and phenotype communities and present the results here.

We first examine the genotype layer-specific communities. To be consistent with the analogous analysis on multiplex disease communities, we take the largest communities with size 10 and greater and consider the top 20 communities. For RR comorbidity, we find that all top-20

communities have higher average RR comorbidity similarity than the background. For the multiplex case, all top-30 communities had higher RR comorbidity than the background as well. Therefore, as a second level of quantification, we compare how significantly higher than the background each case is, measured by $-log(P)$ values of the Mann-Whitney U test for each disease community. We find that RR comorbidity intra-community similarity significances (measured in terms of $-log(P)$) are lower for genotype layer-specific disease communities than multiplex disease communities (23.60 vs 30.72). For MimMiner semantic similarity, we have the same situation where all top-20 communities have higher average MimMiner semantic similarity than the background. For the multiplex case, all top-30 communities also had higher MimMiner semantic similarity than the background. Turning to p-values, we find that MimMiner intra-community similarity significances are lower for genotype layer-specific disease communities than multiplex disease communities (26.79 vs 33.40). For GO:BP similarity, all top-20 communities have higher average GO:BP similarity than the background whereas for the multiplex case, six of the top-30 communities had lower GO:BP similarity than the background. Finally, for gene overlap Jaccard similarity, all top-20 communities have higher average gene overlap Jaccard than the background. For the multiplex case, all top-30 communities had higher gene overlap Jaccard similarity than the background as well. Comparing the p-values, we find that gene overlap Jaccard intra-community similarity significances are significantly higher (two-sided Mann-Whitney U test P=9.47E-05) for genotype layer-specific disease communities than multiplex disease communities (20.28 vs 10.10). Taken together, these results indicate that in terms of phenotypic similarity measures, multiplex disease communities do better than genotype layer-specific disease communities. Meanwhile for molecular similarity measures, the genotype layer communities do better than multiplex communities. We note that the better performance of the genotype layer communities in this case is to be expected since GO:BP similarity and gene overlap are both molecular similarity measures from which the links in this layer are derived.

Second, we examine the phenotype layer-specific communities. We investigate the top-16 disease communities of size 10 and greater. For RR comorbidity, we find that all top-16 communities have higher average RR comorbidity similarity than the background. For the multiplex case, all top-30 communities had higher RR comorbidity than the background as well. The RR comorbidity intra-community similarity significances (measured in terms of $-log(P)$) are higher for phenotype layer-specific disease communities than multiplex disease communities (35.60 vs 30.72), although this difference is not significant (two-sided Wilcoxon Rank Sum test (P=0.10). For MimMiner semantic similarity, once again, all top-16 phenotype layer-specific communities have higher average MimMiner semantic similarity than the background. For the multiplex case, all top-30 communities also had higher MimMiner semantic similarity than the background. The MimMiner intra-community similarity significances are lower for phenotype layer-specific disease communities than multiplex disease communities (28.55 vs 33.40). For GO:BP similarity, five top-16 phenotype layer-specific communities have lower average GO:BP similarity than the background whereas for the multiplex case, six of the top-30 communities had lower GO:BP similarity than the background. The GO:BP intra-community similarity significances are significantly lower (two-sided Wilcoxon Rank Sum test P=0.02) for pheotype layer-specific disease communities than multiplex disease communities (11.06 vs 28.85). For gene overlap Jaccard similarity, all top-16 communities have higher average gene overlap Jaccard than the background. For the multiplex case, all top-30 communities had higher gene overlap Jaccard similarity than the background as well. Comparing the p-values, we find that gene overlap Jaccard intra-community similarity significances are significantly lower (two-sided Wilcoxon Rank Sum test P=0.00055) for phenotype layer-specific disease communities than multiplex disease communities (2.57 vs 10.10). Taken together, these results indicate that multiplex disease communities

do comparably with or better than phenotype layer-specific communities for phenotypic similarity measures, and significantly better than phenotype layer-specific communities for molecular similarity measures. The results of this section are summarized in Fig. S17. Overall, these analyses point towards the cooperative effect of the multiplex disease network where the multiplex disease communities compensate for the lack of molecular similarity in the phenotype layer communities and the lack of phenotypic similarity in the genotype layer communities, offering a good compromise between the two aspects.

Mean(-log(P))	RR comorbidity	MimMiner	GO:BP	Gene Overlap
Multiplex	30.72	33.40	28.85	10.10
Genotype	23.60	26.79	49.04	20.28
Phenotype	35.60	28.55	11.06	2.57

Figure S17: The comparison of multiplex disease communities with genotype and phenotype layer-specific disease communities according to the four similarity measures. Green values indicate the measures for which multiplex communities have higher intra-community similarity than the respective single layer communities whereas red values indicate measures for which multiplex communities have lower intra-community similarity than the respective single layer communities.

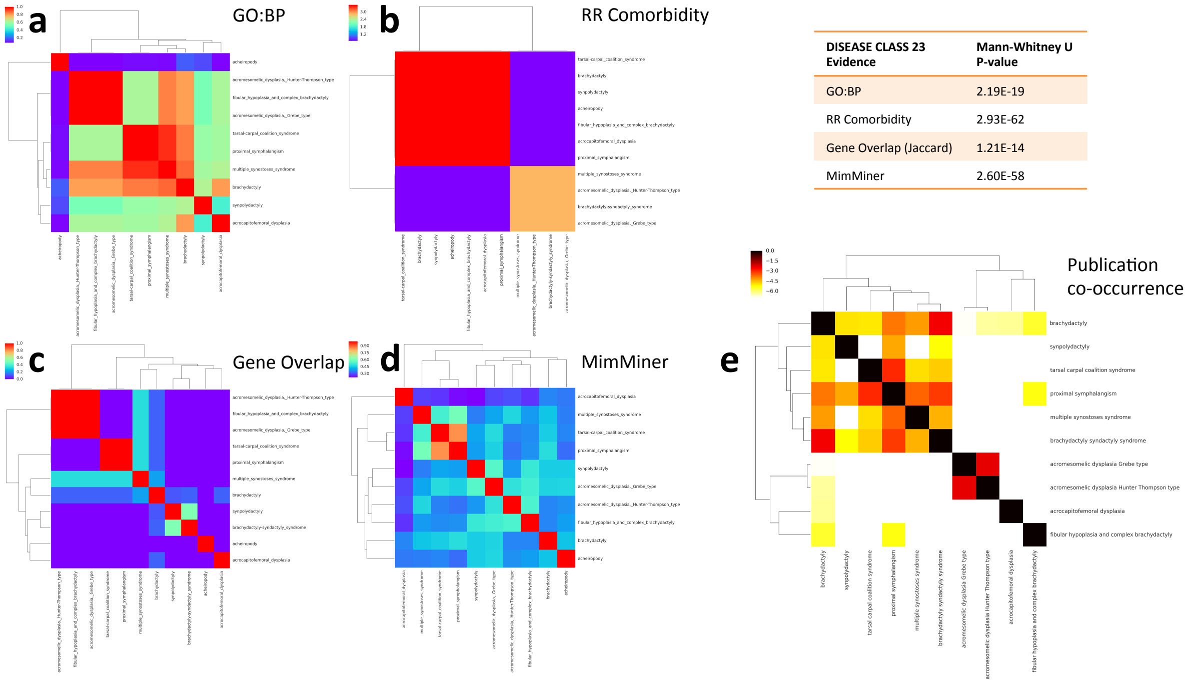

DISEASE CLASS 23 Evidence	Mann-Whitney U P-value
GO:BP	2.19E-19
RR Comorbidity	2.93E-62
Gene Overlap (Jaccard)	1.21E-14
MimMiner	2.60E-58

Figure S18: **Multiplex Disease Community 23.** Disease Community 23 is characterized by rare skeletal abnormalities. **(a)** GO: Biological Process similarity heatmap, where similarity scores range between 0 and 1. **(b)** Relative risk (RR) comorbidity heatmap, where the colors represent the logarithm of the RR values. **(c)** Gene overlap, quantified by the Jaccard index. **(d)** MimMiner phenotype semantic similarity heatmap, where similarity scores range between 0 and 1. **(e)** PubMed literature co-occurrence heatmap and representative network, where the number in each cell denotes the number of publications that have the co-occurence of the queried keywords. The color of each cell represents the Jaccard index and a redder cell means higher literature co-occurrence weighted by the total number of publications.

11 Assessing the clarity of boundaries of multiplex disease communities

As a means to demonstrate the lack of edges between communities, we turn to PubAtlas publication co-occurrence and test whether or not the high publication co-occurrence of intra-layer disease pairs (see Figure 4 in the main text and Supplementary Figures S18 and S24) is accompanied by a lack of publication co-occurrence for inter-layer disease pairs. As proof of concept, we first compare the two communities presented in the main text, disease community 8 and 23, and plot the publication co-occurrence heatmap between these two communities. As expected, we find that there are few publications between the diseases of these two communities (see Fig. S19), where the publication co-occurrence is measured by the Jaccard index J. Overall, the distributions of log(J) of intra-community links for disease community 8 and disease community 23 (mean= -11.45 and -11.87, respectively) are significantly higher than the inter-community links between disease community 8 and disease community 23 (mean= -16.32) (p-value= $3.92E-12$ and $2.47E-07$, respectively). In order to generalize this observation to all disease communities discussed, we plot the pairwise publication co-occurrence heatmap for all communities (Fig. S20). By inspecting Fig. S20, we see that the multiplex communities (along the diagonal) have higher publication co-occurrence similarity within themselves than with other communities, the only exceptions (lines "repeated" in other rows/columns) being the communities connected together with "bridge diseases," which belong to more than one community. When we plot the intra- and inter-community publication co-occurrence Jaccard indices for the top 30 largest communities, we see that there are only two instances where the inter-community co-occurrence was higher than intra-community (diagonal values), i.e. the diagonal is the highest of its respective row (Fig. S21). Among these top 30 largest disease communities, all have higher mean intra-community publication co-occurrence compared to the background and 25 of them are significant (two-sided Mann-Whitney U test). We note that the few "dark" off-diagonal values, even though they have a similar shade of color to the diagonal values, are still of lower co-occurrence by several folds due to the logarithmic scale of the heatmap. Furthermore, we performed the same analysis for layer-specific communities in order to assess their community boundaries. Phenotype communities, which are based on shared symptoms and therefore expected to be more well-defined and to have clearer boundaries in terms of publication co-occurrence. We found that overall, the publication co-occurrence is higher for off-diagonal elements for phenotype layer communities compared to multiplex communities (Fig. S22a), indicating that multiplex communities offer a more well-defined boundary between disease groups measured in terms of publication co-occurrence. For the genotype-specific communities, the intra- vs. inter-community co-occurrences are similar, however the number of strong off-diagonal similarities similar to the diagonal ones is higher in genotype specific communities, indicating that there are more instances where the boundaries between different disease communities are blurred, compared to the multiplex case (Fig. S22b).

Finally, to probe further into which communities are relatively more convoluted, we compare, for each community, its intra-community publication co-occurrence with its inter-community publication co-occurrence with the other 29 communities, and plot the -log(P) values (Fig. S23). We find that although almost all intra-community co-occurrences are higher than inter-community ones (see Fig. S21), a few of them are not significant for some communities (especially communities 3, 7, 17 and 27). This could be due to the low number of publications for these diseases and therefore insufficient statistics.

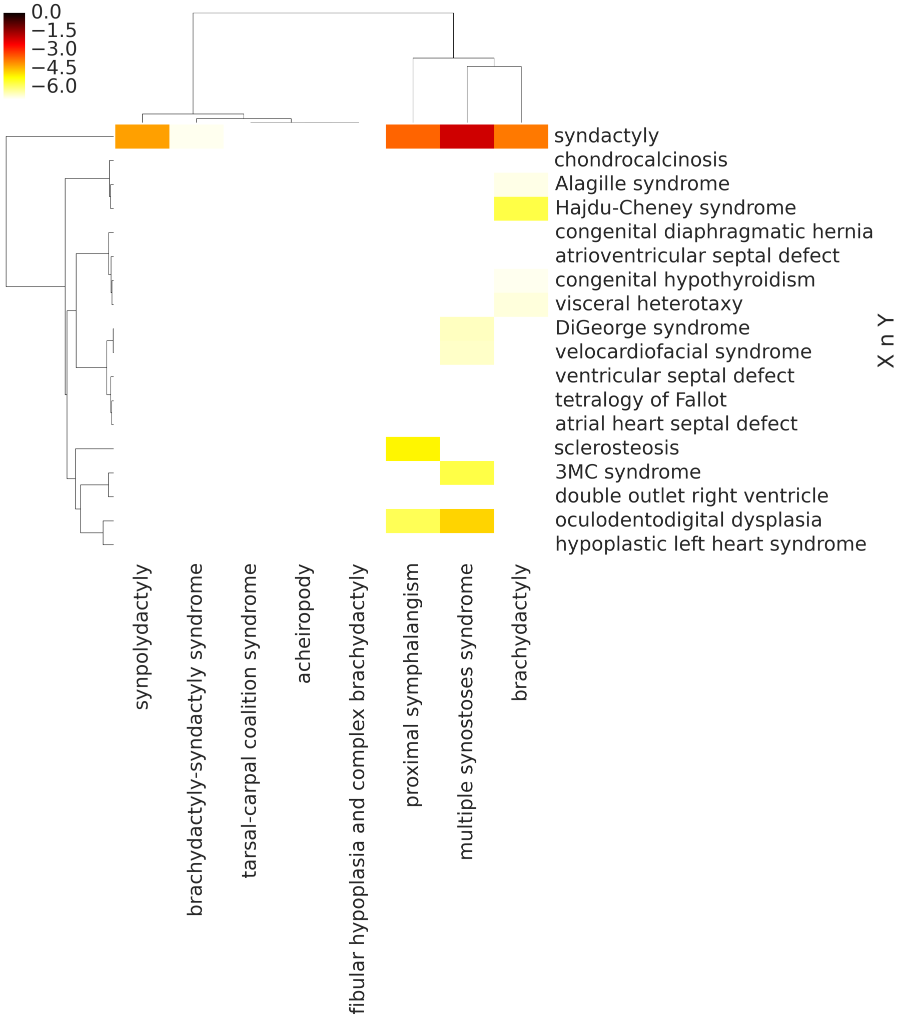

Figure S19: The pairwise publication co-occurrence matrix of all diseases in disease community 8 versus disease community 23.

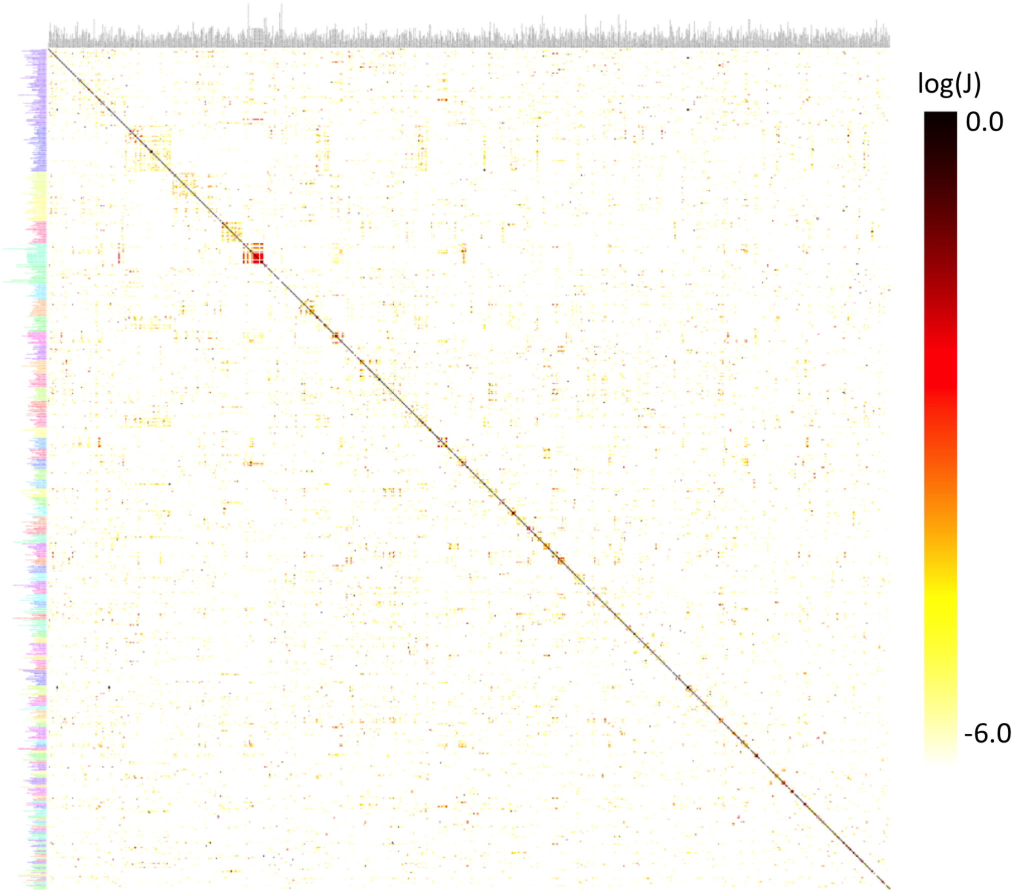

Figure S20: The pairwise publication co-occurrence matrix of all diseases in multiplex disease communities, color coded, ordered in decreasing size.

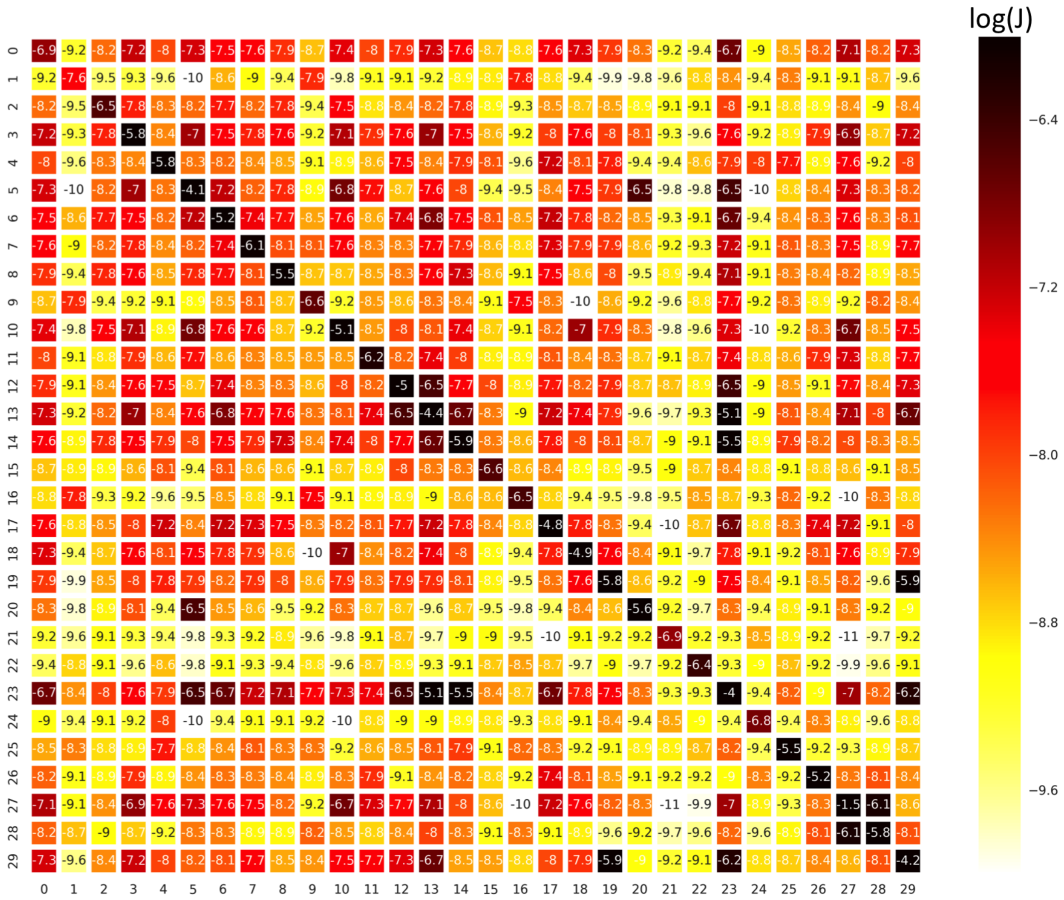

Figure S21: The logarithm of the publication co-occurrence Jaccard indices for the intra- and inter-community diseases in the top 30 multiplex disease communities. Darker color indicates higher publication co-occurrence.

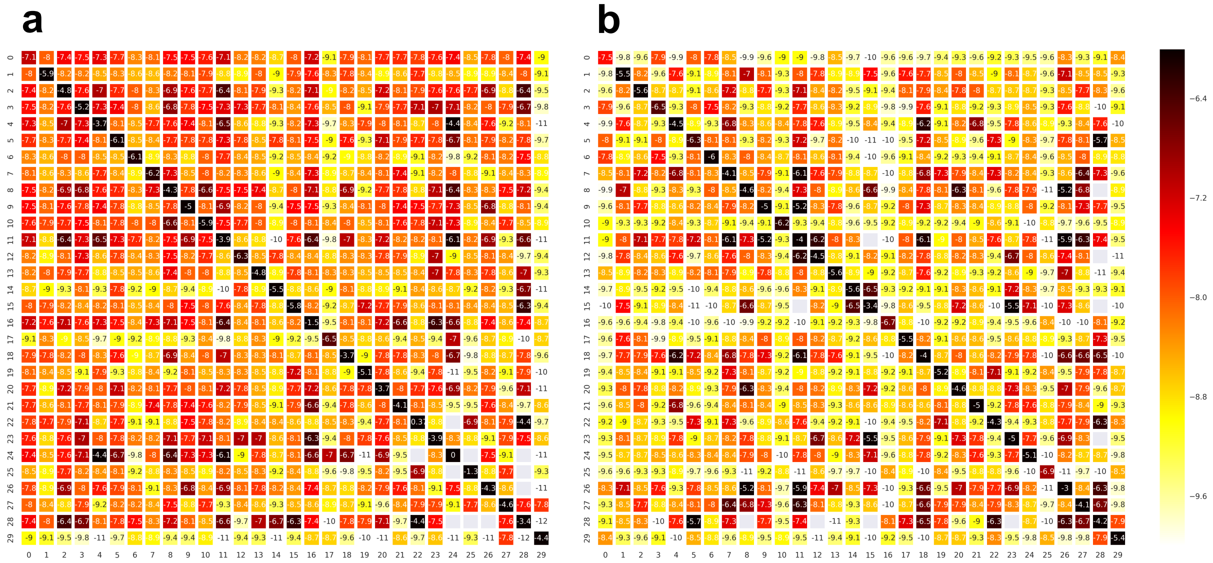

Figure S22: (a) The logarithm of the publication co-occurrence Jaccard indices for the intra- and inter-community diseases in the top 30 phenotype layer disease communities. (b) The logarithm of the publication co-occurrence Jaccard indices for the intra- and inter-community diseases in the top 30 genotype layer disease communities. Darker color indicates higher publication co-occurrence.

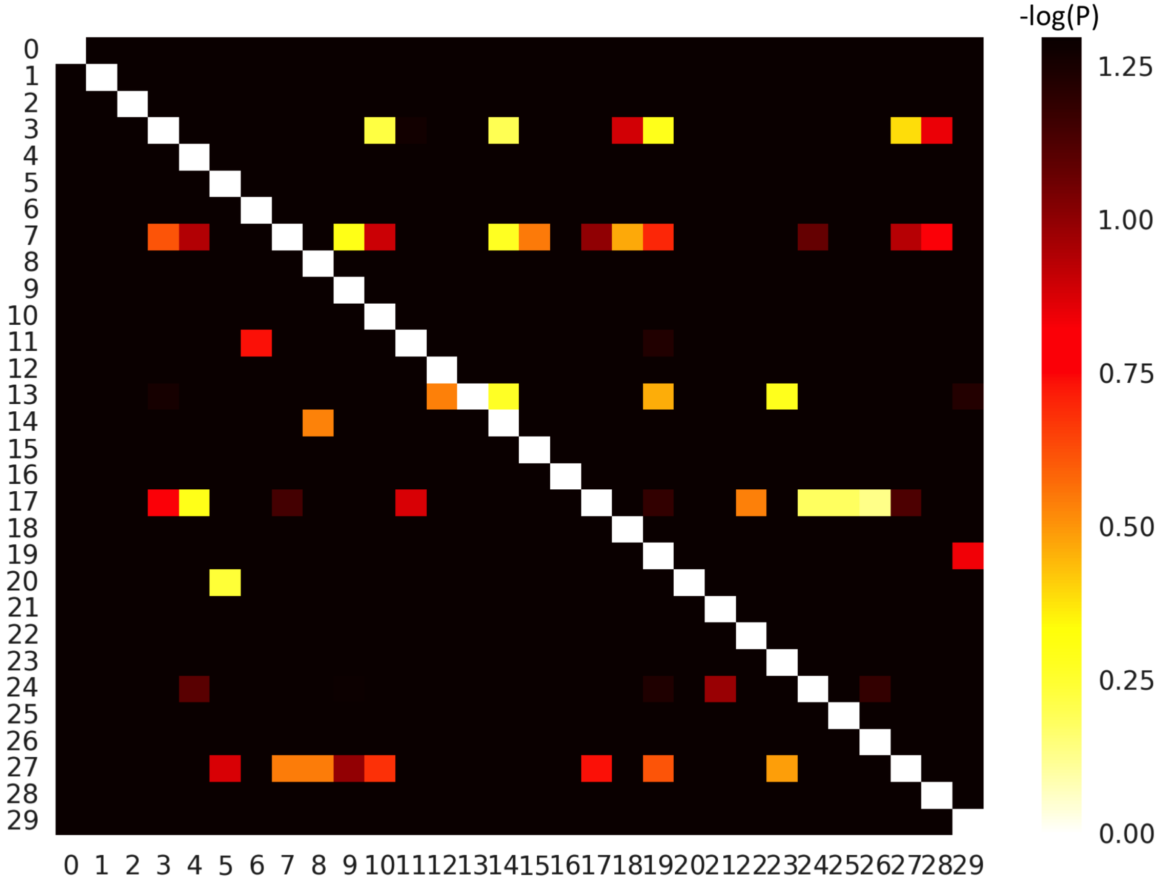

Figure S23: Comparison of intra- and inter-community publication co-occurrences in terms of significance (-log(P) values). In each row, the publication co-occurrence of the community denoted by the row index is compared against all other communities. Any color other than black denotes insignificant ($P > 0.05$, two-sided Kolmogorov-Smirnov test).

12 Preparation of the GWAS dataset

To build the GWAS multiplex, we follow the same procedure as in the main text, with the only difference being the disease-gene bipartite network, i.e. the genotype layer. For the GWAS multiplex, we use the Disease-Connect database [2], and limit our use to the GWAS evidence disease-gene associations. This results in a multiplex network consisting of 113 diseases, divided into 16 multiplex disease communities (Supplementary Table 2).

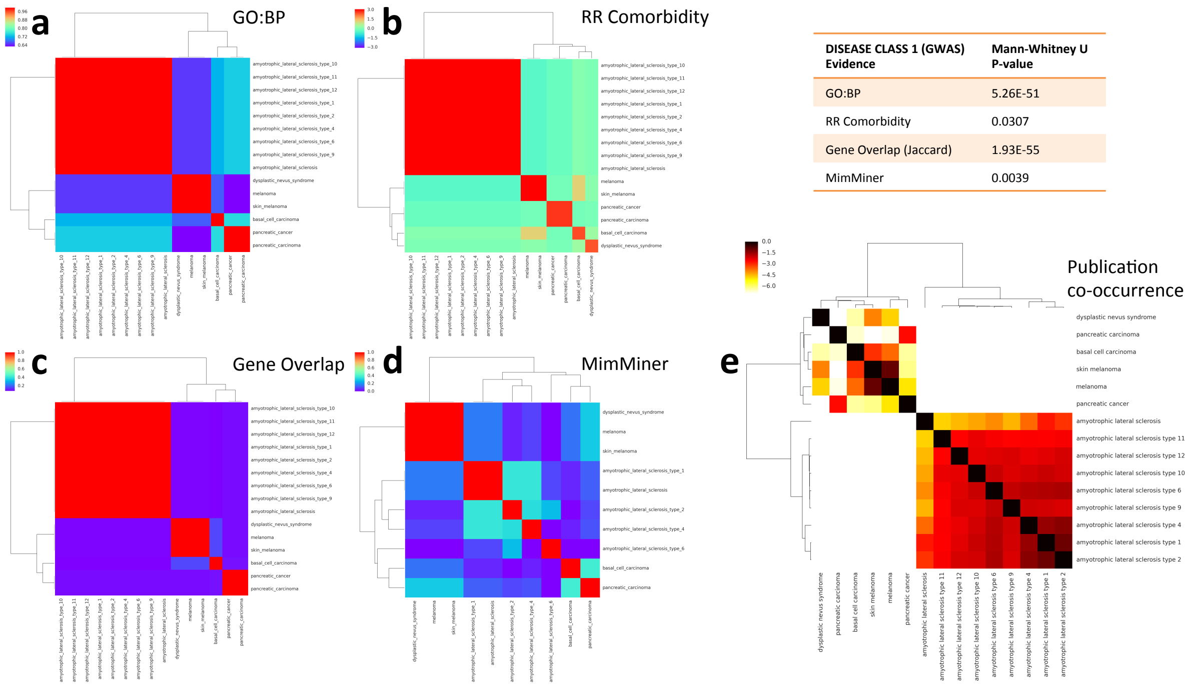

Figure S24: **GWAS Multiplex Disease Community 1.** Disease Community 1 in the GWAS dataset is characterized by ALS types and cancers. **(a)** GO: Biological Process similarity heatmap, where similarity scores range between 0 and 1. **(b)** Relative risk (RR) comorbidity heatmap, where the colors represent the logarithm of the RR values. **(c)** Gene overlap, quantified by the Jaccard index. **(d)** MimMiner phenotype semantic similarity heatmap, where similarity scores range between 0 and 1. **(e)** PubMed literature co-occurrence heatmap and representative network, where the number in each cell denotes the number of publications that have the co-occurence of the queried keywords. The color of each cell represents the Jaccard index and a redder cell means higher literature co-occurrence weighted by the total number of publications.

13 Average gene similarity of disease pairs

To compare the average gene overlap between the genotype, phenotype and overlap network, we calculate the average Jaccard index of overlap over all the edges in these networks. This results in $\langle J \rangle = 0.007$ for the phenotype network, : $\langle J \rangle = 0.208$ for the genotype network, and $\langle J \rangle = 0.252$ for the overlap network (Fig. S25, blue circles). Then to compare these values to random expectation, we generate ensembles of networks with the same degree distribution in each layer separately and calculate the resulting randomized J values. $\langle J \rangle_{rand} = 0.007 \pm 0.001$ for the

phenotype network, $\langle J \rangle_{rand} = 0.011 \pm 0.002$ for the genotype network and $\langle J \rangle_{rand} = 0.011 \pm 0.006$ for the overlap network (Fig. S25, boxplots).

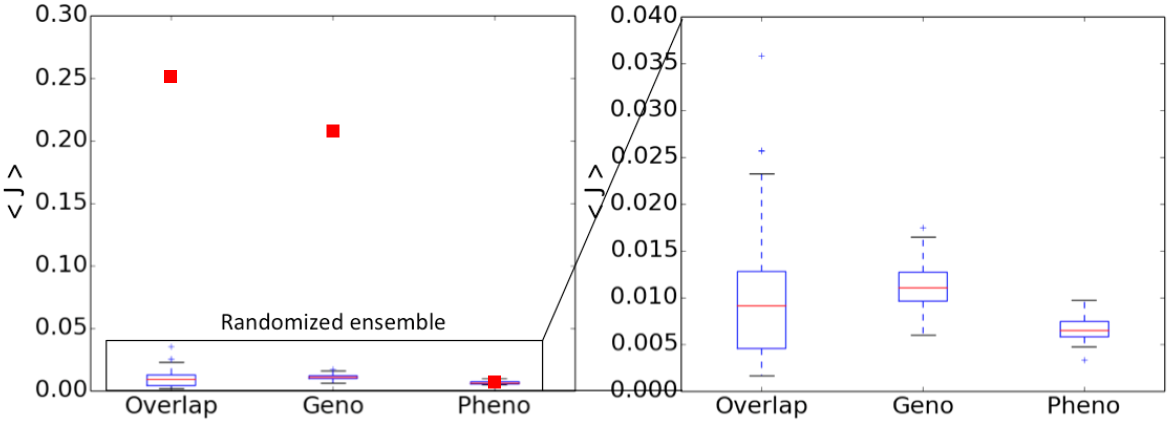

Figure S25: (Left) The average gene similarity of disease pairs in the genotype and phenotype layers, and the overlap network (red squares) against random expectation (boxplots) demonstrates the combined effect of the two sources of information (gene and symptom) on the multiplex construct of the edge overlap layer. (Right) Zoom-in detail of random expectation boxplots. Boxplots represent minimum (Q1 - 1.5 IQR), first quartile (Q1), median, third quartile (Q3), and maximum (Q3 + 1.5 IQR)

References

[1] Mucha, P.J., Richardson, T., Macon, K., Porter, M.A. and Onnela, J.P., 2010. Community structure in time-dependent, multiscale, and multiplex networks. science, 328(5980), pp.876-878.

[2] Liu CC, Tseng YT, Li W, Wu CY, Mayzus I, Rzhetsky A, Sun F, Waterman M, Chen JJ, Chaudhary PM, Loscalzo J. DiseaseConnect: a comprehensive web server for mechanism-based disease-disease connections. Nucleic acids research. 2014 Jul 1;42(W1):W137-46.

Supplemental Table 1		
**Community No.**	**Size**	**Diseases**
0	91	cutis laxa, syndromic intellectual disability, combined oxidative phosphorylation deficiency, congenital muscular dystrophy, maple syrup urine disease, craniosynostosis, hereditary spastic paraplegia, congenital disorder of glycosylation type II, lissencephaly, congenital disorder of glycosylation type I, Albright s hereditary osteodystrophy, Bamforth-Lazarus syndrome, Borjeson-Forssman-Lehmann syndrome, Bowen-Conradi syndrome, Canavan disease, Charcot-Marie-Tooth disease type X, Danon disease, Ellis-Van Creveld syndrome, Farber lipogranulomatosis, Greig cephalopolysyndactyly syndrome, Hartnup disease, Joubert syndrome, L-2-hydroxyglutaric aciduria, Laron syndrome, Larsen syndrome, MASA syndrome, Marshall-Smith syndrome, Perrault syndrome, Saethre-Chotzen syndrome, Sandhoff disease, Seckel syndrome, X-linked ichthyosis, X-linked sideroblastic anemia with ataxia, acrofrontofacionasal dysostosis, acromesomelic dysplasia, Hunter-Thompson type, alpha thalassemia, alpha-mannosidosis, argininosuccinic aciduria, beta-mannosidosis, centronuclear myopathy, cerebrotendinous xanthomatosis, congenital disorder of glycosylation, creatine transporter deficiency, dyskeratosis congenita, erythrokeratodermia variabilis, fibrodysplasia ossificans progressiva, galactosemia, glycogen storage disease II, glycogen storage disease III, hemolytic-uremic syndrome, hydrocephalus, hyperargininemia, hyperlysinemia, isovaleric acidemia, methylmalonic aciduria and homocystinuria type cblD, mevalonic aciduria, microcephaly, microphthalmia, monilethrix, mucolipidosis, mucopolysaccharidosis II, mucosulfatidosis, multiple system atrophy, myotonic dystrophy type 1, non-syndromic X-linked intellectual disability, olivopontocerebellar atrophy, orotic aciduria, peroxisomal acyl-CoA oxidase deficiency, piebaldism, popliteal pterygium syndrome, propionic acidemia, pseudohypoparathyroidism, pseudopseudohypoparathyroidism, sclerosteosis, spinocerebellar ataxia, syndromic X-linked intellectual disability, thalassemia, tyrosinemia type II, Friedreich ataxia, MHC class II deficiency, Matthew-Wood syndrome, premature ovarian failure, hypospadias, amyotrophic lateral sclerosis type 13, attention deficit hyperactivity disorder, triosephosphate isomerase deficiency, congenital ichthyosis, congenital nystagmus, frontotemporal dementia, pontocerebellar hypoplasia type 6, spinocerebellar ataxia type 5
1	54	ovarian cancer, dyskeratosis congenita, piebaldism, LADD syndrome, Peutz-Jeghers syndrome, Noonan syndrome, Costello syndrome, seborrheic keratosis, DNA ligase IV deficiency, LEOPARD syndrome, Li-Fraumeni syndrome, Lynch syndrome, colorectal cancer, Rubinstein-Taybi syndrome, cardiofaciocutaneous syndrome, achondroplasia, autoimmune lymphoproliferative syndrome, breast cancer, colon carcinoma, familial adenomatous polyposis, hemophagocytic lymphohistiocytosis, leprosy, lung small cell carcinoma, mastocytosis, urticaria pigmentosa, pilomatrixoma, pancreatic carcinoma, retinoblastoma, trilateral retinoblastoma, seminoma, testicular cancer, testicular germ cell cancer, stomach cancer, acute myeloid leukemia, Birt-Hogg-Dube syndrome, lung cancer, epidermal nevus, urinary bladder cancer, gastrointestinal stromal tumor, multiple myeloma, cervix carcinoma, thanatophoric dysplasia, adrenocortical carcinoma, choroid plexus papilloma, hepatocellular carcinoma, malignant glioma, osteosarcoma, Proteus syndrome, ataxia telangiectasia, non-Hodgkin lymphoma, embryonal rhabdomyosarcoma, von Hippel-Lindau disease, papillary renal cell carcinoma, juvenile polyposis syndrome

2	30	Andersen-Tawil syndrome, Robinow syndrome, long QT syndrome, Becker muscular dystrophy, Cockayne syndrome, Duchenne muscular dystrophy, Emery-Dreifuss muscular dystrophy, Liddle syndrome, carnitine palmitoyltransferase II deficiency, hemochromatosis, hypertrophic cardiomyopathy, scapuloperoneal myopathy, supravalvular aortic stenosis, Brugada syndrome, atrial heart septal defect, familial atrial fibrillation, short QT syndrome, distal muscular dystrophy, dilated cardiomyopathy, familial partial lipodystrophy, Jervell-Lange Nielsen syndrome, posterior polar cataract, hyperaldosteronism, sick sinus syndrome, sudden infant death syndrome, progeria, arrhythmogenic right ventricular cardiomyopathy, Wolff-Parkinson-White syndrome, restrictive cardiomyopathy, rippling muscle disease
3	29	Walker-Warburg syndrome, nemaline myopathy, Aicardi-Goutieres syndrome, Gauchers disease, Meier-Gorlin syndrome, coenzyme Q10 deficiency disease, congenital generalized lipodystrophy, congenital myasthenic syndrome, muscular dystrophy-dystroglycanopathy, osteopetrosis, multiple system atrophy, Axenfeld-Rieger syndrome, craniometaphyseal dysplasia, Barth syndrome, limb-girdle muscular dystrophy, paroxysmal nocturnal hemoglobinuria, Ullrich congenital muscular dystrophy, oculopharyngeal muscular dystrophy, Fukuyama congenital muscular dystrophy, cytochrome-c oxidase deficiency disease, cystinuria, visceral heterotaxy, von Willebrands disease, inclusion body myositis, glycogen storage disease XV, pontocerebellar hypoplasia type 2A, pontocerebellar hypoplasia type 4, neutral lipid storage disease, situs inversus
4	26	Bothnia retinal dystrophy, cone-rod dystrophy, retinitis pigmentosa, Hirschsprungs disease, Waardenburgs syndrome, Jervell-Lange Nielsen syndrome, Usher syndrome, Leber congenital amaurosis, congenital stationary night blindness, persistent hyperplastic primary vitreous, striatonigral degeneration, achromatopsia, blue cone monochromacy, Stargardt disease, bestrophinopathy, fundus albipunctatus, myopia, cone dystrophy, bradyopsia, cataract, posterior polar cataract, hereditary night blindness, gyrate atrophy, vitelliform macular dystrophy, Aland Island eye disease, partial central choroid dystrophy
5	25	amyotrophic lateral sclerosis type 4, amyotrophic lateral sclerosis, amyotrophic neuralgia, essential tremor, Crouzon syndrome, amyotrophic lateral sclerosis type 1, inclusion body myopathy with Paget disease of bone and frontotemporal dementia, Gerstmann-Straussler-Scheinker syndrome, Niemann-Pick disease, Picks disease, amyotrophic lateral sclerosis type 10, gangliosidosis GM1, amyotrophic lateral sclerosis type 11, amyotrophic lateral sclerosis type 12, amyotrophic lateral sclerosis type 14, amyotrophic lateral sclerosis type 15, amyotrophic lateral sclerosis type 16, amyotrophic lateral sclerosis type 17, amyotrophic lateral sclerosis type 18, amyotrophic lateral sclerosis type 19, amyotrophic lateral sclerosis type 6, amyotrophic lateral sclerosis type 8, amyotrophic lateral sclerosis type 9, amyotrophic lateral sclerosis type 2, primary open angle glaucoma
6	23	autosomal recessive non-syndromic intellectual disability, Warburg micro syndrome, autosomal dominant non-syndromic intellectual disability, infantile cerebellar-retinal degeneration, Andersen-Tawil syndrome, Cornelia de Lange syndrome, Rothmund-Thomson syndrome, Bartter disease, Noonan syndrome, Opitz-GBBB syndrome, acromesomelic dysplasia, Maroteaux type, sialuria, Fanconi syndrome, Lesch-Nyhan syndrome, Weill-Marchesani syndrome, asphyxiating thoracic dystrophy, glycogen storage disease IX, intrahepatic cholestasis, mitochondrial complex V (ATP synthase) deficiency, nuclear type 1, pontocerebellar hypoplasia type 2E, inclusion body myopathy with Paget disease of bone and frontotemporal dementia, juvenile myelomonocytic leukemia, Sotos syndrome

7	19	ACTH-secreting pituitary adenoma, Blau syndrome, Chediak-Higashi syndrome, Clouston syndrome, Hailey-Hailey disease, Jobs syndrome, MHC class I deficiency, Papillon-Lefevre disease, Werner syndrome, acrodermatitis enteropathica, beta thalassemia, biotinidase deficiency, cyclic hematopoiesis, hereditary sensory neuropathy, nevoid basal cell carcinoma syndrome, oculocerebrorenal syndrome, port-wine stain, reticular dysgenesis, Hajdu-Cheney syndrome
8	19	3MC syndrome, tetralogy of Fallot, oculodentodigital dysplasia, Alagille syndrome, sclerosteosis, syndactyly, craniometaphyseal dysplasia, atrial heart septal defect, DiGeorge syndrome, Hajdu-Cheney syndrome, congenital hypothyroidism, visceral heterotaxy, ventricular septal defect, double outlet right ventricle, velocardiofacial syndrome, hypoplastic left heart syndrome, atrioventricular septal defect, chondrocalcinosis, congenital diaphragmatic hernia
9	18	Bannayan-Riley-Ruvalcaba syndrome, Cowden disease, Peutz-Jeghers syndrome, xeroderma pigmentosum, endometrial carcinoma, VACTERL association, basal ganglia calcification, hypospadias, familial meningioma, dysplastic nevus syndrome, melanoma, skin melanoma, follicular thyroid carcinoma, head and neck squamous cell carcinoma, prostate cancer, Kennedys disease, androgen insensitivity syndrome, photosensitive trichothiodystrophy
10	17	centronuclear myopathy, Brown-Vialetto-Van Laere syndrome, Charcot-Marie-Tooth disease type 4, pontocerebellar hypoplasia type 1B, Charcot-Marie-Tooth disease type 2, Charcot-Marie-Tooth disease intermediate type, Charcot-Marie-Tooth disease type 1, distal hereditary motor neuropathy, distal muscular dystrophy, Gamstorp-Wohlfart syndrome, Krabbe disease, Tangier disease, metachromatic leukodystrophy, Charcot-Marie-Tooth disease type 3, focal segmental glomerulosclerosis, brachyolmia, Guillain-Barre syndrome
11	17	3-M syndrome, achondrogenesis type II, spondylocostal dysostosis, Leigh disease, Walker-Warburg syndrome, acrodysostosis, atelosteogenesis, holoprosencephaly, hypopituitarism, osteogenesis imperfecta, otospondylomegaepiphyseal dysplasia, pre-eclampsia, Bjornstad syndrome, Bruck syndrome, multiple sclerosis, cytochrome-c oxidase deficiency disease, schneckenbecken dysplasia
12	16	Meckel syndrome, Joubert syndrome, renal-hepatic-pancreatic dysplasia, Bardet-Biedl syndrome, asphyxiating thoracic dystrophy, autistic disorder, erythropoietic protoporphyria, generalized epilepsy with febrile seizures plus, nephrotic syndrome, thrombophilia, triple-A syndrome, nephronophthisis, Senior-Loken syndrome, glycogen storage disease IV, hypermethioninemia, orofaciodigital syndrome
13	16	Aarskog-Scott syndrome, FG syndrome, Kallmann syndrome, Meckel syndrome, Ohdo syndrome, Prader-Willi syndrome, Roberts syndrome, SC phocomelia syndrome, Simpson-Golabi-Behmel syndrome, Troyer syndrome, cold-induced sweating syndrome, multiple synostoses syndrome, oculodentodigital dysplasia, tarsal-carpal coalition syndrome, synpolydactyly, periventricular nodular heterotopia
14	16	nemaline myopathy, distal arthrogryposis, Antley-Bixler syndrome, Carpenter syndrome, Loeys-Dietz syndrome, brachydactyly, Bethlem myopathy, syndactyly, CHARGE syndrome, hydrolethalus syndrome, Down syndrome, multiple intestinal atresia, persistent fetal circulation syndrome, Marfan syndrome, Smith-McCort dysplasia, proximal symphalangism

15	16	cutis laxa, hemolytic-uremic syndrome, Nijmegen breakage syndrome, supravalvular aortic stenosis, Sensenbrenner syndrome, focal segmental glomerulosclerosis, acute lymphocytic leukemia, age related macular degeneration, gallbladder disease, orofacial cleft, systemic lupus erythematosus, complement component 2 deficiency, complement factor I deficiency, chronic myeloid leukemia, retinal drusen, complement component 3 deficiency
16	15	holoprosencephaly, nevoid basal cell carcinoma syndrome, Fanconis anemia, nephrotic syndrome, Frasier syndrome, breast cancer, hereditary breast ovarian cancer, desmoplastic medulloblastoma, medulloblastoma, pancreatic cancer, pancreatic carcinoma, Denys-Drash syndrome, malignant mesothelioma, nephroblastoma, basal cell carcinoma
17	13	Clouston syndrome, erythrokeratodermia variabilis, Baraitser-Winter syndrome, autosomal dominant nonsyndromic deafness, autosomal recessive nonsyndromic deafness, Usher syndrome, angioma serpiginosum, renal tubular acidosis, keratoconus, nasopharynx carcinoma, Bart-Pumphrey syndrome, dentin dysplasia, dentinogenesis imperfecta
18	13	mucopolysaccharidosis III, Scheie syndrome, acute porphyria, atypical teratoid rhabdoid tumor, mucopolysaccharidosis I, spastic quadriplegia, aspartylglucosaminuria, fucosidosis, homocystinuria, mucopolysaccharidosis VI, mucopolysaccharidosis, omodysplasia, periventricular nodular heterotopia
19	12	episodic ataxia, benign familial infantile epilepsy, idiopathic generalized epilepsy, clubfoot, familial hemiplegic migraine, familial meningioma, generalized dystonia, optic atrophy, porencephaly, specific language impairment, juvenile myoclonic epilepsy, alternating hemiplegia of childhood
20	12	Alzheimers disease, cerebral amyloid angiopathy, hemochromatosis, progressive supranuclear palsy, Picks disease, amyotrophic lateral sclerosis type 17, familial visceral amyloidosis, frontotemporal dementia, sea-blue histiocyte syndrome, porphyria cutanea tarda, variegate porphyria, hidradenitis suppurativa
21	12	Hermansky-Pudlak syndrome, thrombophilia, DiGeorge syndrome, schizophrenia, homocystinuria, factor V deficiency, cerebrovascular disease, essential hypertension, alcohol dependence, prothrombin deficiency, factor XIII deficiency, panic disorder
22	11	type 1 diabetes mellitus, Donohue Syndrome, cataract, maturity-onset diabetes of the young, type 2 diabetes mellitus, nonpapillary renal cell carcinoma, renal cell carcinoma, hyperinsulinemic hypoglycemia, Wolfram syndrome, diabetic ketoacidosis, pancreatic agenesis
23	11	multiple synostoses syndrome, tarsal-carpal coalition syndrome, acromesomelic dysplasia, Hunter-Thompson type, brachydactyly, synpolydactyly, acheiropody, fibular hypoplasia and complex brachydactyly, acrocapitofemoral dysplasia, proximal symphalangism, brachydactyly-syndactyly syndrome, acromesomelic dysplasia, Grebe type
24	11	X-linked hypophosphatemia, arterial calcification of infancy, oculocutaneous albinism, spondyloarthropathy, obesity, congenital nystagmus, ocular albinism, pilomatrixoma, pseudoxanthoma elasticum, asthma, migraine
25	11	ABCD syndrome, Hirschsprungs disease, Waardenburgs syndrome, renal agenesis, neuroblastoma, phaeochromocytoma, MEN2A, MEN2B, familial medullary thyroid carcinoma, paraganglioma, alveolar rhabdomyosarcoma

26	11	osteogenesis imperfecta, 46 XX gonadal dysgenesis, osteoporosis, osteopetrosis, Bruck syndrome, Caffey disease, guanidinoacetate methyltransferase deficiency, type I Ehlers-Danlos syndrome, Pagets disease of bone, exudative vitreoretinopathy, rickets
27	10	Ehlers-Danlos syndrome progeroid type, Refsum disease, achondrogenesis type IB, infantile refsum disease, rhizomelic chondrodysplasia punctata, spondyloepiphyseal dysplasia congenita, aceruloplasminemia, fragile X-associated tremor/ataxia syndrome, X-linked chondrodysplasia punctata, thyroid hormone resistance syndrome
28	10	46 XX gonadal dysgenesis, 46 XY gonadal dysgenesis, congenital adrenal insufficiency, ovarian cancer, fragile X syndrome, azoospermia, premature ovarian failure, fragile X-associated tremor/ataxia syndrome, ovarian hyperstimulation syndrome, blepharophimosis, ptosis, and epicanthus inversus syndrome
29	9	lissencephaly, generalized epilepsy with febrile seizures plus, Dravet Syndrome, Ohtahara syndrome, infantile epileptic encephalopathy, infantile onset spinocerebellar ataxia, benign familial infantile epilepsy, benign neonatal seizures, Partington syndrome
30	9	pre-eclampsia, Kallmann syndrome, Saethre-Chotzen syndrome, acrocephalosyndactylia, Antley-Bixler syndrome, Beare-Stevenson cutis gyrata syndrome, CHARGE syndrome, Crouzon syndrome, Pfeiffer syndrome
31	9	atelosteogenesis, Larsen syndrome, Beare-Stevenson cutis gyrata syndrome, diastrophic dysplasia, achondrogenesis type IB, multiple epiphyseal dysplasia, Boomerang dysplasia, pseudoachondroplasia, osteoarthritis
32	9	hereditary sensory neuropathy, congenital bile acid synthesis defect, Troyer syndrome, hereditary spastic paraplegia, hereditary lymphedema, congenital generalized lipodystrophy, Pelizaeus-Merzbacher disease, primary pulmonary hypertension, pulmonary venoocclusive disease
33	9	ABCD syndrome, Hermansky-Pudlak syndrome, cyclic hematopoiesis, Down syndrome, Glanzmanns thrombasthenia, thrombocytopenia, severe congenital neutropenia, congenital dyserythropoietic anemia, Wiskott-Aldrich syndrome
34	8	Cockayne syndrome, leukocyte adhesion deficiency, Menkes disease, severe congenital neutropenia, UV-sensitive syndrome, pyridoxine-refractory autosomal recessive sideroblastic anemia, pyruvate decarboxylase deficiency, autoimmune lymphoproliferative syndrome
35	8	hydrolethalus syndrome, VACTERL association, IMAGe syndrome, Johanson-Blizzard syndrome, Koolen de Vries syndrome, N syndrome, Townes-Brocks syndrome, acrocallosal syndrome
36	8	achondrogenesis type II, otospondylomegaepiphyseal dysplasia, Stickler syndrome, osteochondritis dissecans, spondyloepiphyseal dysplasia congenita, Legg-Calve-Perthes Disease, alkaptonuria, spondyloepimetaphyseal dysplasia, Strudwick type
37	8	Marshall-Smith syndrome, Baller-Gerold syndrome, Beckwith-Wiedemann syndrome, Nijmegen breakage syndrome, Pallister-Hall syndrome, IMAGe syndrome, Sotos syndrome, Silver-Russell syndrome

38	7	neuronal ceroid lipofuscinosis, Parkinsons disease, progressive myoclonus epilepsy, essential tremor, familial juvenile hyperuricemic nephropathy, basal ganglia calcification, biotin-responsive basal ganglia disease
39	7	multiple epiphyseal dysplasia, achondroplasia, acrocapitofemoral dysplasia, brachyolmia, cleidocranial dysplasia, spondyloepiphyseal dysplasia with congenital joint dislocations, hereditary multiple exostoses
40	7	non-syndromic X-linked intellectual disability, Coffin-Lowry syndrome, Rett syndrome, Smith-Lemli-Opitz syndrome, cutaneous porphyria, fragile X syndrome, cardiofaciocutaneous syndrome
41	7	autosomal dominant type IV Ehlers-Danlos syndrome, Ehlers-Danlos syndrome, arterial tortuosity syndrome, cornea plana, type III Ehlers-Danlos syndrome, type VI Ehlers-Danlos syndrome, vesicoureteral reflux
42	7	Blau syndrome, Kaposis sarcoma, inflammatory bowel disease, ulcerative colitis, juvenile rheumatoid arthritis, sea-blue histiocyte syndrome, sarcoidosis
43	7	Adams-Oliver syndrome, Fanconis anemia, campomelic dysplasia, hereditary lymphedema, Caffey disease, Diamond-Blackfan anemia, LEOPARD syndrome
44	7	popliteal pterygium syndrome, hypohidrotic ectodermal dysplasia, Bloch-Sulzberger syndrome, tooth agenesis, Van der Woude syndrome, orofacial cleft, tooth and nail syndrome
45	7	Aicardi-Goutieres syndrome, MHC class II deficiency, systemic lupus erythematosus, leprosy, rheumatoid arthritis, dyschromatosis symmetrica hereditaria, complement component 4a deficiency
46	6	benign neonatal seizures, central core myopathy, choreatic disease, dihydropyrimidine dehydrogenase deficiency, hypokalemic periodic paralysis, malignant hyperthermia
47	6	Bloch-Sulzberger syndrome, Costello syndrome, epidermodysplasia verruciformis, focal dermal hypoplasia, lipoid proteinosis, seborrheic keratosis
48	6	Bjornstad syndrome, KBG syndrome, Nager syndrome, Netherton syndrome, Renpenning syndrome, autosomal dominant type IV Ehlers-Danlos syndrome
49	6	3-Methylcrotonyl-CoA carboxylase deficiency, fatal infantile encephalocardiomyopathy, nephrogenic diabetes insipidus, polycystic liver disease, Bardet-Biedl syndrome, myopia
50	6	Dowling-Degos disease, advanced sleep phase syndrome, azoospermia, dyschromatosis universalis hereditaria, reticulate acropigmentation of Kitamura, vesicoureteral reflux
51	6	Donohue Syndrome, familial partial lipodystrophy, Fabry disease, Frasier syndrome, Kaposis sarcoma, nail-patella syndrome
52	6	ACTH-secreting pituitary adenoma, Albrights hereditary osteodystrophy, pseudohypoparathyroidism, pseudopseudohypoparathyroidism, growth hormone secreting pituitary adenoma, prolactinoma
53	6	acrodysostosis, Carney complex, neurilemmomatosis, striatonigral degeneration, primary pigmented nodular adrenocortical disease, papillary thyroid carcinoma

54	6	hypopituitarism, microphthalmia, GABA aminotransferase deficiency, Klippel-Feil syndrome, coloboma, Matthew-Wood syndrome
55	6	congenital myasthenic syndrome, Loeys-Dietz syndrome, hypokalemic periodic paralysis, esophagus squamous cell carcinoma, familial Mediterranean fever, hyperkalemic periodic paralysis
56	5	congenital adrenal hyperplasia, hypogonadism, glucocorticoid-remediable aldosteronism, hyperaldosteronism, primary pigmented nodular adrenocortical disease
57	5	Kahrizi syndrome, congenital hypothyroidism, urofacial syndrome, hypochromic microcytic anemia, obesity
58	5	Alport syndrome, LADD syndrome, Riley-Day syndrome, Sjogren-Larsson syndrome, acrocephalosyndactylia
59	5	Greig cephalopolysyndactyly syndrome, Pallister-Hall syndrome, acheiropody, hamartoma of hypothalamus, polydactyly
60	5	Huntingtons disease, Tay-Sachs disease, abetalipoproteinemia, episodic ataxia, pseudo-TORCH syndrome
61	5	Charcot-Marie-Tooth disease type X, Arts syndrome, renal-hepatic-pancreatic dysplasia, X-linked nonsyndromic deafness, sensorineural hearing loss
62	5	syndromic X-linked intellectual disability, Asperger syndrome, autistic disorder, Rett syndrome, Angelman syndrome
63	5	Alpers syndrome, deafness dystonia syndrome, pontocerebellar hypoplasia type 8, pontocerebellar hypoplasia type 9, Jensen syndrome
64	5	celiac disease, Gerstmann-Straussler-Scheinker syndrome, multiple sclerosis, fatal familial insomnia, Creutzfeldt-Jakob disease
65	4	Gauchers disease, Parkinsons disease, metachromatic leukodystrophy, Lewy body dementia
66	4	methylmalonic acidemia, methylmalonic aciduria and homocystinuria type cblC, methylmalonic aciduria and homocystinuria type cblF, transcobalamin II deficiency
67	4	Allan-Herndon-Dudley syndrome, celiac disease, hypohidrotic ectodermal dysplasia, type 1 diabetes mellitus
68	4	adult spinal muscular atrophy, intermediate spinal muscular atrophy, Werdnig-Hoffmann disease, juvenile spinal muscular atrophy
69	4	Crigler-Najjar syndrome, Gilbert syndrome, Rh deficiency syndrome, congenital nonspherocytic hemolytic anemia
70	4	neurilemmomatosis, juvenile myelomonocytic leukemia, atypical teratoid rhabdoid tumor, neurofibromatosis
71	4	Baraitser-Winter syndrome, PSPH deficiency, glycogen storage disease VI, non-syndromic intellectual disability
72	4	Laron syndrome, Tangier disease, coronary artery disease, familial hypercholesterolemia
73	4	Liddle syndrome, bronchiectasis, cystic fibrosis, Camurati-Engelmann disease
74	4	Rothmund-Thomson syndrome, Baller-Gerold syndrome, Li-Fraumeni syndrome, rapadilino syndrome
75	4	familial adenomatous polyposis, pachyonychia congenita, epidermolytic hyperkeratosis, nonepidermolytic palmoplantar keratoderma

76	4	familial lipoprotein lipase deficiency, hyperlipoproteinemia type V, familial combined hyperlipidemia, familial hypertriglyceridemia
77	4	Alexander disease, cartilage-hair hypoplasia, anauxetic dysplasia, spondyloepimetaphyseal dysplasia, Missouri type
78	3	megaloblastic anemia, pontocerebellar hypoplasia type 10, pontocerebellar hypoplasia type 2D
79	3	hemorrhagic thrombocythemia, myelofibrosis, polycythemia vera
80	3	CLONE OF congenital afibrinogenemia, congenital afibrinogenemia, familial visceral amyloidosis
81	3	Kartagener syndrome, primary ciliary dyskinesia, dyslexia
82	3	Pfeiffer syndrome, Rubinstein-Taybi syndrome, glycine encephalopathy
83	3	Brooke-Spiegler syndrome, Finnish type amyloidosis, progressive supranuclear palsy
84	3	autosomal dominant polycystic kidney, polycystic kidney disease, autosomal recessive polycystic kidney
85	3	gangliosidosis GM1, mucopolysaccharidosis IV, mucopolysaccharidosis IX
86	3	coloboma, aniridia, coloboma of optic nerve
87	3	peripheral primitive neuroectodermal tumor, retinoblastoma, trilateral retinoblastoma
88	3	factor V deficiency, factor XII deficiency, hereditary angioedema
89	3	pontocerebellar hypoplasia type 2A, pontocerebellar hypoplasia type 4, pontocerebellar hypoplasia type 5
90	3	hypotrichosis, peeling skin syndrome, alopecia universalis
91	3	acute intermittent porphyria, fatal familial insomnia, hereditary coproporphyria
92	3	Asperger syndrome, blepharospasm, attention deficit hyperactivity disorder
93	3	Weill-Marchesani syndrome, Marfan syndrome, primary congenital glaucoma
94	3	primary cutaneous amyloidosis, transthyretin amyloidosis, carpal tunnel syndrome
95	3	common variable immunodeficiency, idiopathic pulmonary fibrosis, immunoglobulin alpha deficiency
96	3	multinodular goiter, papillary thyroid carcinoma, pleuropulmonary blastoma
97	3	hereditary multiple exostoses, chondrosarcoma, extraskeletal myxoid chondrosarcoma
98	2	ARC syndrome, congenital bile acid synthesis defect
99	2	Bruton-type agammaglobulinemia, agammaglobulinemia
100	2	Bethlem myopathy, Ullrich congenital muscular dystrophy
101	2	craniosynostosis, parietal foramina
102	2	Axenfeld-Rieger syndrome, iridogoniodysgenesis syndrome

103	2	Lynch syndrome, Muir-Torre syndrome
104	2	acrokeratosis verruciformis, keratosis follicularis
105	2	Moyamoya disease, thoracic aortic aneurysm
106	2	hyperparathyroidism, parathyroid carcinoma
107	2	intrahepatic cholestasis, cholecystitis
108	2	open-angle glaucoma, primary open angle glaucoma
109	2	sialuria, inclusion body myositis
110	2	beta thalassemia, sickle cell anemia
111	2	branchiootic syndrome, branchiootorenal syndrome
112	2	hemophagocytic lymphohistiocytosis, lymphoproliferative disease
113	2	DNA ligase IV deficiency, bloom syndrome
114	2	erythropoietic protoporphyria, X-linked sideroblastic anemia
115	2	lung small cell carcinoma, pleomorphic adenoma
116	2	Alagille syndrome, Schmid metaphyseal chondrodysplasia
117	2	blue cone monochromacy, red-green color blindness
118	2	complement factor I deficiency, xanthinuria
119	2	complement component 6 deficiency, complement component 7 deficiency
120	2	carbamoyl phosphate synthetase I deficiency disease, citrullinemia
121	2	branchiooculofacial syndrome, cleft palate
122	2	Kawasaki disease, holocarboxylase synthetase deficiency
123	2	hereditary elliptocytosis, hereditary spherocytosis
124	2	combined T cell and B cell immunodeficiency, gamma chain deficiency
125	2	Sugio-Kajii syndrome, trichorhinophalangeal syndrome type I
126	2	Gilles de la Tourette syndrome, trichotillomania
127	2	Papillon-Lefevre disease, aggressive periodontitis

**Supplemental Table 2**		
**Community No**	**Size**	**Diseases**
0	34	Alzheimers disease, Behcets disease, Fuchs endothelial dystrophy, Graves disease, Hodgkins lymphoma, IgA glomerulonephritis, Kawasaki disease, Parkinsons disease, Wegeners granulomatosis, abdominal aortic aneurysm, age related macular degeneration, alopecia areata, aortic valve stenosis, asthma, atopic dermatitis, breast cancer, celiac disease, cerebrovascular disease, cervix carcinoma, cleft palate, colitis, coronary artery disease, cystic fibrosis, eosinophilic esophagitis, essential tremor, familial abdominal aortic aneurysm, hyperthyroidism, hypotrichosis, inflammatory bowel disease, leprosy, lung small cell carcinoma, malignant glioma, nephroblastoma, panic disorder
1	15	amyotrophic lateral sclerosis type 10, amyotrophic lateral sclerosis type 11, amyotrophic lateral sclerosis type 12, amyotrophic lateral sclerosis type 1, amyotrophic lateral sclerosis type 2, amyotrophic lateral sclerosis type 4, amyotrophic lateral sclerosis type 6, amyotrophic lateral sclerosis type 9, amyotrophic lateral sclerosis, basal cell carcinoma, dysplastic nevus syndrome, melanoma, pancreatic cancer, pancreatic carcinoma, skin melanoma
2	14	leprosy, narcolepsy, osteoporosis, primary biliary cirrhosis, progressive supranuclear palsy, psoriasis, rheumatoid arthritis, spondyloarthropathy, systemic lupus erythematosus, thyrotoxicosis, type 1 diabetes mellitus, type 2 diabetes mellitus, ulcerative colitis, vitiligo
3	14	Pagets disease of bone, endometrial carcinoma, hypokalemic periodic paralysis, idiopathic generalized epilepsy, multiple sclerosis, myasthenia gravis, myopia, nephrotic syndrome, obesity, ovarian cancer, prostate cancer, renal cell carcinoma, tuberous sclerosis, uterine fibroid
4	6	alpha thalassemia, beta thalassemia, gallbladder disease, hemolytic anemia, sickle cell anemia, thalassemia
5	5	acute leukemia, acute lymphocytic leukemia, age related macular degeneration, gallbladder disease, polycystic ovary syndrome
6	5	IgA glomerulonephritis, alpha thalassemia, autistic disorder, nephrotic syndrome, thalassemia
7	4	colon carcinoma, colorectal cancer, migraine, tetralogy of Fallot
8	4	lymphoproliferative disease, multiple myeloma, sarcoidosis, stomach cancer
9	3	seminoma, testicular cancer, testicular germ cell cancer
10	3	neuroblastoma, osteosarcoma, peripheral primitive neuroectodermal tumor
11	2	Wegeners granulomatosis, beta thalassemia
12	2	azoospermia, obesity
13	2	hypokalemic periodic paralysis, idiopathic generalized epilepsy
14	1	urinary bladder cancer
15	1	inflammatory bowel disease
